# Dipolar-Coupled Entangled Molecular 4f Qubits

**DOI:** 10.1021/jacs.2c10902

**Published:** 2023-01-25

**Authors:** Bela E. Bode, Edoardo Fusco, Rachel Nixon, Christian D. Buch, Høgni Weihe, Stergios Piligkos

**Affiliations:** † EaStCHEM School of Chemistry, Biomedical Sciences Research Complex, and Centre of Magnetic Resonance, University of St Andrews, North Haugh, St AndrewsKY16 9ST, U.K.; ‡ Department of Chemistry, University of Copenhagen, CopenhagenDK-2100, Denmark

## Abstract

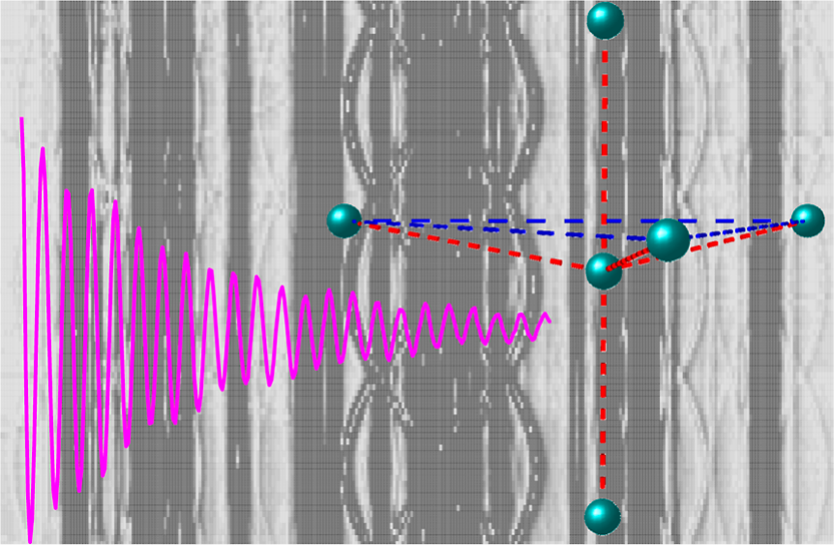

We demonstrate by use of continuous wave- and pulse-electron paramagnetic resonance spectroscopy on oriented single crystals of magnetically dilute Yb^III^ ions in Yb_0.01_Lu_0.99_(trensal) that molecular entangled two-qubit systems can be constructed by exploiting dipolar interactions between neighboring Yb^III^ centers. Furthermore, we show that the phase memory time and Rabi frequencies of these dipolar-interaction-coupled entangled two-qubit systems are comparable to the ones of the corresponding single qubits.

## Introduction

The current emergence of quantum technologies, within the second quantum revolution,^1−3^ is based on the exploitation of genuine quantum properties of matter, such as superposition and entanglement, to develop new technologies such as quantum computing, simulators, communications, sensing, metrology, cryptography, and imaging. In particular, the realization of a general purpose quantum computer^4−8^ (QC) is currently one of the most ambitious technological goals.^9,10^ QCs will outperform classical computers (quantum advantage) for some specific types of computations, such as prime number factorization,^11^ large database search,^12^ or the accurate simulation of quantum many-body systems.^13^ Thus, QCs will transform searching and sharing information and will have a disruptive impact on innovation in materials and chemicals with applications in energy (magnets, batteries, and superconductors), agriculture (efficient and sustainable fertilizers) and biomedicine and biotechnologies.

Very recently, superconducting and photonic quantum processing units (QPUs) were announced to have attained quantum advantage.^14−16^ However, even these very impressive QPUs cannot efficiently address practical problems related to quantum error correction.^17^ Thus, it is anticipated that future general purpose QCs will not be solely based on superconducting or photonic qubits but will require additional, or entirely different, components offering more efficient ways to fight against quantum error.

Molecular magnetic materials offer possibilities to circumvent some of these limitations and therefore constitute a very promising avenue for the next-generation quantum information technology devices.^18−23^ Unlike many other candidates, molecular magnetic materials routinely display many low energy states compatible with the encoding of qubits and even acting as integrated quantum processors, the additional levels providing the capability to expand the dimension of the computational space or to efficiently encode quantum error correction algorithms.^24−34^ The critical parameter for the suitability of such materials for use in quantum information devices is the phase memory time, *T*_m_, reflecting the time for which the state in which information is encoded retains its phase coherence.^35^ Decoherence,^36^ the interaction of the quantum system with its environment, results in loss of superposition and/or entanglement, collapsing the dynamic state of the system to its thermal equilibrium static eigenvectors.

The primary strategy to reduce decoherence in molecular magnetic materials consists in magnetic dilution to reduce magnetic dipolar exchange. Other approaches include isotopic enrichment to modify the nuclear spin composition of the environment or chemically engineered systems displaying magnetic clock transitions.^37^ However, as previously noticed,^37^ there is an apparent intrinsic fundamental contradiction between the need for magnetic dilution in order to preserve the coherent magnetic properties and the need for the individual qubits not to be entirely isolated from each other, in order to allow implementation of two-qubit gates that are necessary for the execution of quantum algorithms via universal sets of single- and coupled-qubit gate operations.^38,39^

## Results and Discussion

Lanthanide (Ln) complexes are a rather unexplored but very interesting class of molecular spin qubits.^20,24,28,29,37,40,41^ Some of us have previously demonstrated that the ground Kramers doublet of the ^2^F_7/2_ ground term of the trigonal Yb(trensal)^42^ is an excellent electronic qubit.^24^ Yb(trensal) is also a prototypical coupled electronic-qubit–nuclear-qudit where efficient quantum error correction algorithms can intrinsically be implemented.^25^ In these previous studies of the coherent electronic properties of Yb(trensal), doped into the isostructural diamagnetic host Lu(trensal) to minimize dipolar decoherence, we noticed that both the continuous wave (c.w.)- and pulse-EPR spectra contained resonances that were not attributable to single-ion ones and were initially assigned as “minority sites characterized by the presence of neighboring Yb^III^ centers”.^24^ We show herein, by use of single-crystal c.w.- and pulse-EPR spectroscopy, that our initial assignment of these lines was correct and demonstrate that magnetic dilution is not incompatible with the implementation of coupled-qubit gates. To this purpose, we probe the coherent magnetic properties of such entangled two-qubit systems by pulse-EPR and demonstrate the ability to coherently drive them. Such molecular entangled two-qubit systems have been only demonstrated for transition metal heterometallic wheels,^43−47^ even presenting clock transitions,^48^ but never for Ln complexes, within the context of molecular materials for quantum information. In this latter case, an example of a family of heterodinuclear Ln complexes exists where two-qubit gates were proposed within the same molecule, by exploiting the difference of g-factors of the two different Ln centers,^40^ and thus not between independent 4f qubits. However, there is a growing number of reports on pulsed-EPR of pairs of Gd^III^ ions, particularly by DEER spectroscopy.^49−51^

**Figure 1:**
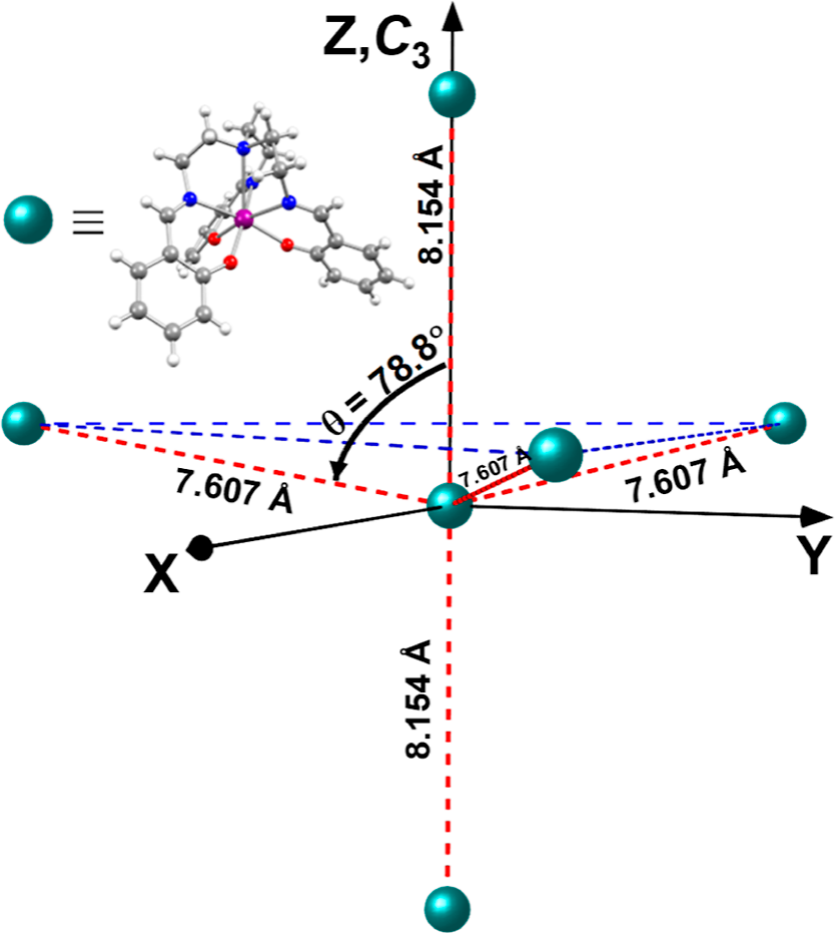
Depiction of first-neighbor sites, within a Cartesian reference frame, in the crystal structure of Yb(trensal), where Yb(trensal) is depicted as a sphere (insert), for simplicity.

Yb(trensal), as other members of the Ln(trensal) family, crystallizes in the *P*3̅*c*1 space group (Table S1) as large pencil-shaped crystals in which the Yb^III^ ion and the apical tertiary amine nitrogen atom define the molecular *C*_3_ axis which is coincident with the crystallographic *C*_3_ axis(Figure S1).^42,52−59^ Two different molecular orientations are found along the trigonal crystallographic axis, corresponding to a relative rotation of two Yb(trensal) molecules by 48° around the *C*_3_ axis (Figure S2). However, the axial nature of Yb^III^ centers in Yb(trensal) imposes that these two different molecular orientations are magnetically equivalent when the applied magnetic field is oriented along the trigonal axis or normal to it. Furthermore, the combination of the *C*_3_ axis and of the inversion center (Figure S3) generates three molecular orientations defining a plane normal to the *C*_3_ axis (Figure 1), where the internuclear Yb^III^-Yb^III^ distance vectors, *R⃗*, between each of the Yb^III^ centers in this plane and the one at the origin, make an angle of θ = 78.8° with the *C*_3_ axis. These five, in total, sites define the first-neighbor sites in the crystal structure of Yb(trensal).

**Figure 2:**
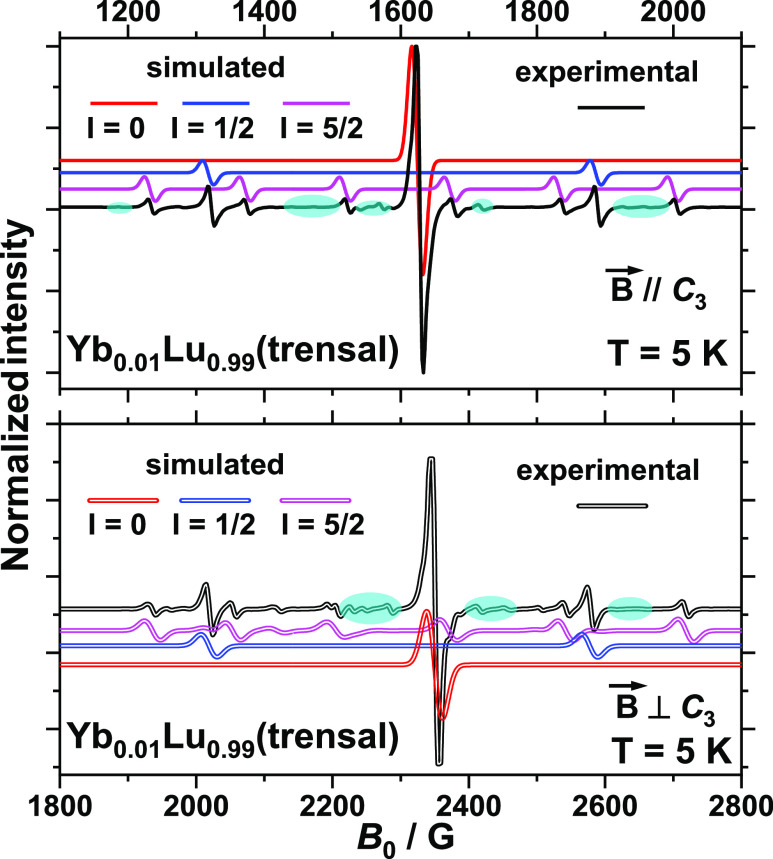
X-band c.w.-EPR spectrum of an oriented single crystal of **1** with the magnetic field along (top) or perpendicular to (bottom) the *C*_3_ axis and simulations of the contributions from various isotopes of Yb. The highlighted areas indicate lines originating from dipolar interaction-coupled neighboring Yb(III) centers.

**Figure 3:**
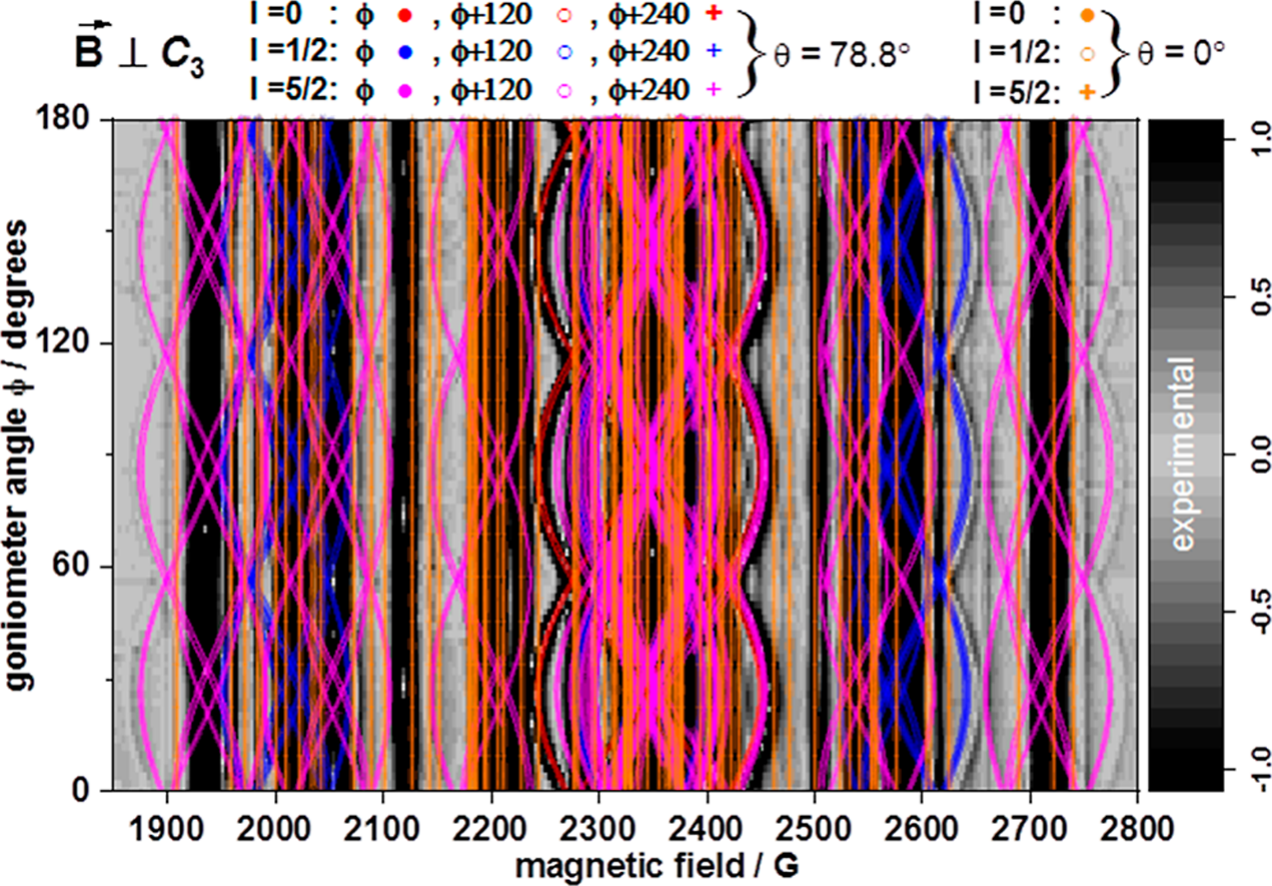
Angular variation of the X-band c.w.-EPR spectrum of **1** in the plane perpendicular to the *C*_3_ axis and at 15 K, with θ being the angle of the internuclear distance vector *R⃗* and the *C*_3_ axis (experiment in shades of gray and simulations of bands arising from dipolar interactions in color).

In previous c.w.- and pulse-EPR and NMR studies of Yb(trensal) diluted in Lu(trensal),^24,25,42^ we accurately determined the static parameters of the Hamiltonian of Yb(trensal), both within ligand field^42^ and effective ground doublet^24,25^ models. In particular, for a ground Kramers doublet effective electronic spin-half model, the Hamiltonian has the form
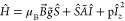
1including electron Zeeman, hyperfine, and nuclear quadrupolar terms, with μ_B_ being the electron Bohr magneton, *B⃗* being the external magnetic field of magnitude *B*_0_,  being the g-tensor, *Ã* being the hyperfine interaction tensor, and *p* being the nuclear quadrupolar parameter. The natural composition of Yb encompasses the isotopes ^168,170–174,176^Yb of which ^171^Yb (14%) and ^173^Yb (16%) possess a nuclear spin (*I* = 1/2 and 5/2, respectively). The determined parameters for the trigonal, non-interacting Yb^III^ centers in Yb(trensal) were *g*_⊥_ = 2.93, *g*_||_ = 4.29, ^171^*A*_⊥_ = 0.0748 cm^–1^, ^171^*A*_||_ = 0.111 cm^–1^, ^173^*A*_⊥_ = −0.0205 cm^–1^, ^173^*A*_||_ = −0.02993 cm^–1^, and ^173^*p* = −0.0022 cm^–1^.^24,25,42^ These parameters are obtained by modeling contributions from isolated Yb^III^ centers to the EPR spectra and not from sites where two Yb^III^ centers are first neighbors. Thus, these parameters alone cannot reproduce a number of weaker lines of the c.w.- and pulse-EPR spectra, such as, for example, the ones illustrated in the highlighted regions of the c.w.-EPR spectra of Figure 2, for Yb(trensal) diluted in the isostructural Lu(trensal) at the 1% level [Yb_0.01_Lu_0.99_(trensal), **1**, as determined by ICP-MS (Supporting Information section)]. In c.w.-EPR, the intensity ratio of the “single-ion” versus “coupled” lines is given by the probability ratio that Yb^III^ occupies one or both of two neighboring sites, this ratio being 1% for **1**. Given the axial nature of the Hamiltonian describing Yb^III^ in **1**, the “single-ion” resonances should present no angular dependence for magnetic field orientations normal to the *C*_3_ axis (intense lines in Figures 2 and 3 and S4–S8). Similarly, resonances originating from coupled Yb^III^ centers where both centers are located on the *C*_3_ axis (Figure 1) should also present no angular variation for orientations of the magnetic field normal to the *C*_3_ axis (Figures 3 and S8), assuming that the interaction between the Yb^III^ centers is purely of magnetic dipole character. The magnetic dipole interaction between two Yb^III^ centers is given by^60^
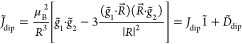
2with *R⃗* being the internuclear distance vector, 1̃ being the unit matrix, and *J*_dip_ and  being the zeroth- and second-order contributions to the spin–spin interaction, respectively. Thus, in the case that *R⃗* is along the *C*_3_ axis (Figure 1) and *B⃗* is normal to the *C*_3_ axis, given the axial nature of *g̃* of Yb^III^ in **1**, the interaction energy between the two Yb^III^ centers is constant for any orientation of *B⃗* normal to the *C*_3_ axis (Figures 3 and S8). In contrast, when *R⃗* is at an angle θ = 78.8° (or for any θ ≠ 0°) with the *C*_3_ axis (Figure 1), rotating *B⃗* in the plane normal to the *C*_3_ axis results in an angular dependence of the resonance fields that for **1** should present a 60° periodicity (Figures 3 and S5–S7). The magnetic dipole interaction tensors for two interacting Yb^III^ centers for which the internuclear vector *R⃗* is along the *C*_3_ axis (, ) or at an angle θ = 78.8° with the *C*_3_ axis () are
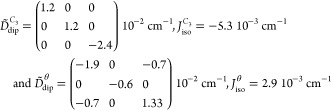


The total Hamiltonian, , for a pair of interacting Yb^III^ centers is the sum of Hamiltonians (1) for each center and an interaction Hamiltonian, , including terms relative to the interaction energy expressed in (2). Thus
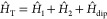
3with . Diagonalization of the matrix representation of (3) with the previously mentioned single-ion and exchange parameters in the basis spanned by a Yb^III^ center with *I*_1_ = 0 interacting with a Yb^III^ center with *I*_2_ = 0, 1/2, or 5/2 (matrix dimension of 4, 8, or 24, respectively) located on the *C*_3_ axis or at an angle θ = 78.8° to it results in the reproduction of all the observed resonances of **1** (Figure 3 and S5–S8) that cannot be attributed to isolated Yb^III^ sites. It is remarkable that the detailed angular dependence of the observed single-crystal spectra for a rotation of the external magnetic field in the plane normal to the *C*_3_ axis can be obtained by considering only the point dipole magnetic interaction and with the use of no free fit parameters.

In previous studies,^24^ we observed that the echo-detected-field-swept (EDFS) X-band single-crystal pulse-EPR spectra of Yb_0.07_Lu_0.93_(trensal) displayed relative intensities for the resonances originating from coupled Yb^III^ sites with respect to the single-ion ones that were similar to the corresponding relative intensities of the c.w. spectra (Figure S9). Thus, since the EDFS spectra are weighted by the echo intensity, this points to *T*_m_s for the coupled-site resonances similar to the ones of the single-ion ones. This observation prompted us to study in detail the coherent properties of these coupled-site resonances in single crystals of **1**. These single crystals display narrow enough linewidths for the purpose of this study and originate from the same batch as the ones where the unambiguous attribution of the nature of these lines by c.w.-EPR has been performed. X-band EDFS spectra were recorded on oriented single crystals of **1**, with *B⃗* in the plane normal to the *C*_3_ axis or parallel to it and the microwave field  perpendicular to *B⃗* (Figure S10), by the use of a Hahn echo^61^ pulse sequence (π/2–τ–π–τ–echo). All the observed features in these EDFS spectra can be assigned to resonances from single-ion and coupled Yb^III^ sites, in full consistency with the c.w.-EPR spectra.

**Figure 4:**
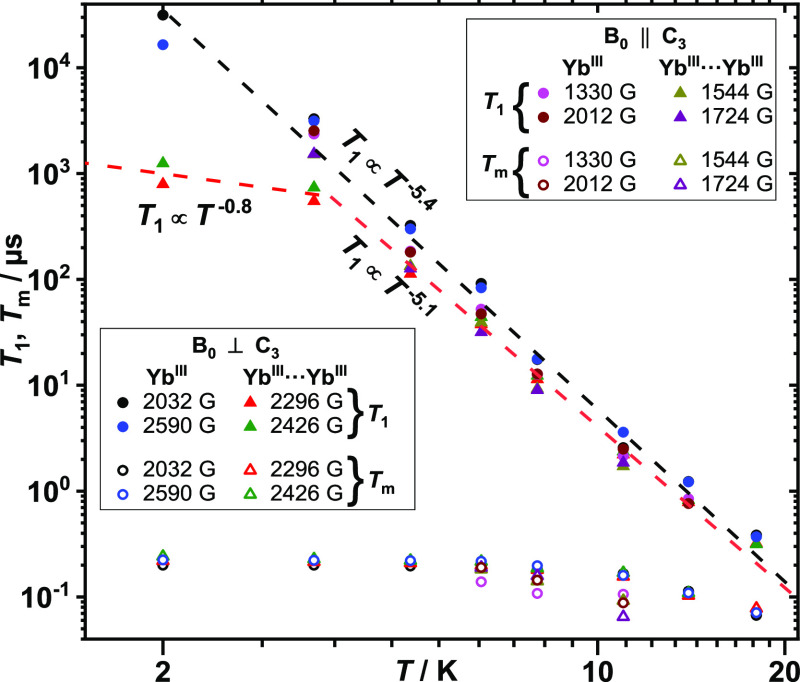
*T*_1_ (solid) and *T*_m_ (hollow) for single-ion (circles) and coupled (triangles) Yb(III) sites at different temperatures and magnetic fields for *B*_0_||*C*_3_ and *B*_0_⊥*C*_3_.

The coherent spin dynamics of single- or coupled-qubit sites, represented by eigenstates involved in the observed resonances, was probed by their corresponding *T*_m_. For coupled sites, resonances originating from two interacting Yb^III^ centers with *I* = 0 were targeted because of their higher intensities resulting from the abovementioned natural abundances of Yb isotopes. *T*_m_s were determined by monitoring the time evolution of the intensity of the Hahn echo, obtained by the sequence π/2−τ–π–τ–echo, at magnetic fields corresponding to the resonance field of a given transition for *B⃗* parallel or perpendicular to the *C*_3_ axis (Figures 4 and S12, S14). The obtained *T*_m_s show that the Yb^III^-coupled sites display similar coherent quantum dynamics to the single-ion ones (Tables S3 and S5). Furthermore, the determined *T*_m_s are essentially temperature-independent up to about 20 K, whereupon they become *T*_1_-limited (Figure 4). The longitudinal relaxation times *T*_1_, corresponding to each of these resonances, were determined by the inversion recovery sequence π*–*τ’*–*π/2−τ–π–τ–echo (Figures 4, S11, S13 and Tables S2 and S4). Interestingly, the temperature dependence of *T*_1_ shows that at higher temperatures, Raman processes drive *T*_1_ relaxation (*T*_1_ α *T*^–5^) for both single-ion and coupled Yb^III^ sites. At lower temperatures, the temperature dependence of *T*_1_ for coupled sites suggests a phonon-bottlenecked direct process (*T*_1_ α *T*^–2^), while no change in regime is discerned for single-ion sites. These observations are in good agreement with our previous pulse-EPR studies on *T*_1_ of single-ion sites where only Raman processes were shown to be relevant in the investigated temperature range (3.3–20 K).^24^ However, in previous single-crystal SQUID magnetometry *T*_1_ studies of Yb (trensal), we showed that *T*_1_ is governed by a phonon-bottlenecked direct process at low temperatures, while at higher temperatures, there is a regime change to Raman relaxation.^42^ These observations demonstrate the ability of pulse-EPR to provide very detailed deconvoluted information on relaxation dynamics. It has to be noted here, however, that the phonon bottleneck effects observed in the SQUID measurements could also be due to the different crystal sizes or magnetic fields used.

**Figure 5:**
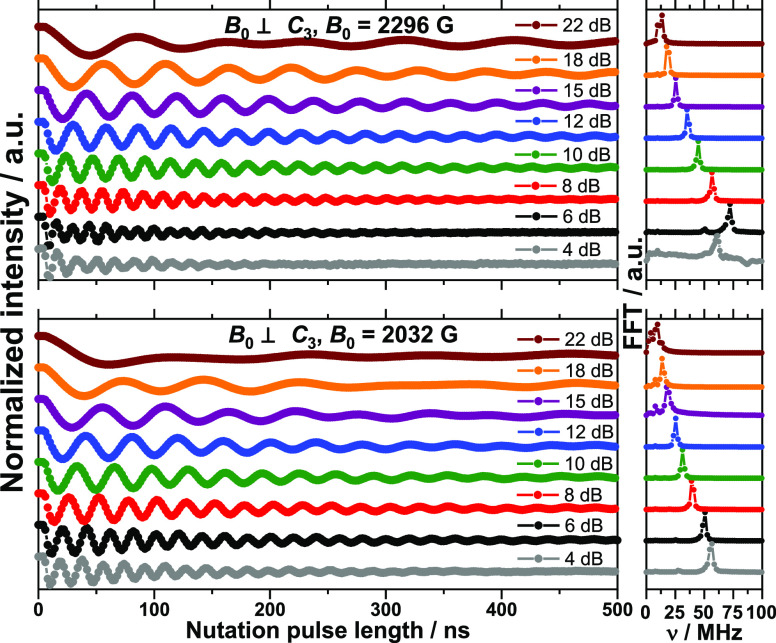
Dipolar exchange-coupled (top) and single-ion (bottom) microwave-power-dependent Rabi oscillations for a crystal of **1** oriented with *B*_0_⊥*C*_3_ (left) and corresponding Fourier transforms (right).

The ability to coherently drive the dynamic states corresponding to each of the observed resonances was probed by transient nutation experiments (Figure 5 and S15–S22). The observed oscillations of the probed echo intensity correspond to Rabi oscillations, as demonstrated by their linear dependence on attenuation and thus magnetic field () amplitude (Figures S23 and S24). The observed Rabi frequencies are of the order of magnitude of tens of MHz, which is in good agreement with the expected ones, given by  which is of the order of 50*g* MHz, with *B*_1_ being the amplitude of the microwave field (of the order of 10 Gauss at zero attenuation), *g* being the *g*-factor of the probed levels, and *h* being the Planck constant. The corresponding gate times (time at the first minimum of the Rabi oscillations) are of the order of 10 ns, thus much shorter than *T*_m_. The observed Rabi frequencies corresponding to single-ion transitions are smaller by a factor of 1.3 (*B⃗*||*C*_3_) to 1.4 (*B⃗*⊥*C*_3_) with respect to resonances attributed to coupled Yb^III^ sites (Tables S6 and S7). This is in good agreement with the theoretical ratio of , corresponding to the ratio of transition matrix elements  for *S* = 1/2 or 1, for the single-ion or coupled Yb^III^ sites, respectively. Thus, the experimentally determined ratio of Rabi frequencies unambiguously confirms the nature of the observed resonances as assigned by c.w.-EPR. More importantly, the observation of Rabi oscillations confirms the possibility to coherently drive the dipolar interaction-coupled sites.

At low magnetic fields (), the eigenvectors for two coupled Yb^III^ sites are of the form
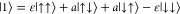

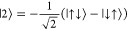

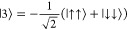

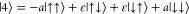
with *a* ≫ ε (Tables S8–S12). While |2⟩ and |3⟩ correspond to the  and  Bell states, respectively, |1⟩ and |4⟩ only resemble, albeit closely, the  and  Bell states, respectively. Such states presenting a high degree of entanglement (maximum in the case of Bell states) are necessary for the implementation of universal quantum gates comprising a series of single-qubit rotations and a two-qubit gate, such as, for example, the controlled NOT gate. The ability to factorize these states to direct product states, such as, for example, |1⟩′ = |↑⟩|↑⟩; |2⟩′ = |↑⟩|↓⟩; |3⟩′ = |↓⟩|↑⟩; and |4⟩′ = |↓⟩|↓⟩, could be implemented by applying the magnetic field to a general orientation where various Yb^III^ sites can differ in the g-value. Another possibility is the application of local electric fields to each independent Yb^III^ site that would affect the g-factor of each site independently, leading to the factorization of the entangled wave functions, as in the case of previously reported heterodinuclear Ln complexes.^38,40,63^

However, the EPR experiments presented herein were performed at high magnetic fields () where the difference in exchange interaction in different spatial directions is very small compared to the applied magnetic field. Thus, the spin–spin interaction becomes effectively isotropic with corresponding eigenvectors (Tables S8–S12) tending to
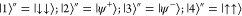
and thus to the eigenvectors of the isotropic exchange. Thus, manipulation of Bell-like states for the systems presented herein is only possible at much lower frequencies than the ones used herein.

In conclusion, we demonstrate herein that magnetic dilution is not inherently contradictory with the construction of molecular entangled two-qubit systems that can be coherently manipulated with Rabi frequencies of the same order of magnitude as the corresponding single qubits.

## Supporting Information Available

The research data supporting this publication can be accessed at University of St Andrews Research Portal: https://doi.org/10.17630/128f8a32-350d-485d-b5a7-3e9cc2b2e9ad. The Supporting Information is available free of charge at https://pubs.acs.org/doi/10.1021/jacs.2c10902.

Experimental details, characterization, and EPR data (PDF)

## References

[ref1] Riedel M. F.; Bloch I.; Debuisschert T.; Wilhelm-Mauch F.; Pruneri V.; Vitanov N. V.; Wehner S.; Calarco T. Europe’s Quantum Flagship is taking off. EuroPhys. News 2018, 49, 30–34. 10.1051/epn/2018506.

[ref2] Atzori M.; Sessoli R. The Second Quantum Revolution: Role and Challenges of Molecular Chemistry. J. Am. Chem. Soc. 2019, 141, 11339–11352. 10.1021/jacs.9b00984. 31287678

[ref3] MacFarlane A. G. J.; Dowling J. P.; Milburn G. J. Quantum technology: the second quantum revolution. Philosophical Transactions of the Royal Society of London Series A: Mathematical, Physical and Engineering Sciences 2003, 361, 1655–1674. 10.1098/rsta.2003.1227 12952679

[ref4] Feynman R. P. Quantum-Mechanical Computers. Found. Phys. 1986, 16, 507–531. 10.1007/bf01886518.

[ref5] Awschalom D.; Samarth N.; Loss D. Semiconductor Spintronics and Quantum Computation; Springer: Berlin, 2002.

[ref6] Barnett S. M. Quantum Information; Oxford University Press: Oxford, 2009, p 16.

[ref7] Preskill J. “Quantum Computing in the NISQ era and beyond”. Quantum 2018, 2, 79 10.22331/q-2018-08-06-79.

[ref8] Gibney E. Physics: Quantum computer quest. Nature 2014, 516, 24–26. 10.1038/516024a. 25471864

[ref9] DiCarlo L.; Reed M. D.; Sun L.; Johnson B. R.; Chow J. M.; Gambetta J. M.; Frunzio L.; Girvin S. M.; Devoret M. H.; Schoelkopf R. J. Preparation and measurement of three-qubit entanglement in a superconducting circuit. Nature 2010, 467, 574–578. 10.1038/nature09416. 20882013

[ref10] Thiele S.; Balestro F.; Ballou R.; Klyatskaya S.; Ruben M.; Wernsdorfer W. Electrically driven nuclear spin resonance in single-molecule magnets. Science 2014, 344, 1135–1138. 10.1126/science.1249802. 24904159

[ref11] Shor P. W. Algorithms for quantum computation: discrete logarithms and factoring. Proceedings 35th Annual Symposium on Foundations of Computer Science, 1994; pp 124–134.

[ref12] Grover L. K. Quantum Mechanics Helps in Searching for a Needle in a Haystack. Phys. Rev. Lett. 1997, 79, 325–328. 10.1103/physrevlett.79.325.

[ref13] Cirac J. I.; Zoller P. Goals and opportunities in quantum simulation. Nat. Phys. 2012, 8, 264–266. 10.1038/nphys2275.

[ref14] Arute F.; et al. Quantum supremacy using a programmable superconducting processor. Nature 2019, 574, 505–510. 10.1038/s41586-019-1666-5. 31645734

[ref15] Wu Y.; et al. Strong Quantum Computational Advantage Using a Superconducting Quantum Processor. Phys. Rev. Lett. 2021, 127, 180501 10.1103/PhysRevLett.127.180501. 34767433

[ref16] Zhong H.-S.; et al. Phase-Programmable Gaussian Boson Sampling Using Stimulated Squeezed Light. Phys. Rev. Lett. 2021, 127, 180502 10.1103/physrevlett.127.180502. 34767431

[ref17] Terhal B. M. Quantum error correction for quantum memories. Rev. Mod. Phys. 2015, 87, 307–346. 10.1103/revmodphys.87.307.

[ref18] Rocha A. R.; García-suárez V. M.; Bailey S. W.; Lambert C. J.; Ferrer J.; Sanvito S. Towards molecular spintronics. Nat. Mater. 2005, 4, 335–339. 10.1038/nmat1349. 15750597

[ref19] Bogani L.; Wernsdorfer W. Molecular spintronics using single-molecule magnets. Nat. Mater. 2008, 7, 179–186. 10.1038/nmat2133. 18297126

[ref20] Gaita-Ariño A.; Luis F.; Hill S.; Coronado E. Molecular spins for quantum computation. Nat. Chem. 2019, 11, 301–309. 10.1038/s41557-019-0232-y. 30903036

[ref21] Sessoli R. All in one. Nat. Phys. 2021, 17, 1192–1193. 10.1038/s41567-021-01382-1.

[ref22] Sessoli R. Tackling the challenge of controlling the spin with electric field. Natl. Sci. Rev. 2020, 8, nwaa267 10.1093/nsr/nwaa267. 34691580 PMC8288386

[ref23] Bonizzoni C.; Ghirri A.; Santanni F.; Atzori M.; Sorace L.; Sessoli R.; Affronte M. Storage and retrieval of microwave pulses with molecular spin ensembles. npj Quantum Information 2020, 6, 68 10.1038/s41534-020-00296-9.

[ref24] Pedersen K. S.; Ariciu A. M.; McAdams S.; Weihe H.; Bendix J.; Tuna F.; Piligkos S. Toward Molecular 4f Single-Ion Magnet Qubits. J. Am. Chem. Soc. 2016, 138, 5801–5804. 10.1021/jacs.6b02702. 27105449

[ref25] Hussain R.; Allodi G.; Chiesa A.; Garlatti E.; Mitcov D.; Konstantatos A.; Pedersen K. S.; De Renzi R.; Piligkos S.; Carretta S. Coherent Manipulation of a Molecular Ln-Based Nuclear Qudit Coupled to an Electron Qubit. J. Am. Chem. Soc. 2018, 140, 9814–9818. 10.1021/jacs.8b05934. 30040890

[ref26] Gimeno I.; Urtizberea A.; Román-Roche J.; Zueco D.; Camón A.; Alonso P. J.; Roubeau O.; Luis F. Broad-band spectroscopy of a vanadyl porphyrin: a model electronuclear spin qudit. Chem. Sci. 2021, 12, 5621–5630. 10.1039/d1sc00564b. 34168797 PMC8179683

[ref27] Carretta S.; Zueco D.; Chiesa A.; Gómez-León Á.; Luis F. A perspective on scaling up quantum computation with molecular spins. Appl. Phys. Lett. 2021, 118, 240501 10.1063/5.0053378.

[ref28] Jenkins M. D.; Duan Y.; Diosdado B.; García-Ripoll J. J.; Gaita-Ariño A.; Giménez-Saiz C.; Alonso P. J.; Coronado E.; Luis F. Coherent manipulation of three-qubit states in a molecular single-ion magnet. Phys. Rev. B 2017, 95, 064423 10.1103/physrevb.95.064423.

[ref29] Martínez-Pérez M. J.; Cardona-Serra S.; Schlegel C.; Moro F.; Alonso P. J.; Prima-García H.; Clemente-Juan J. M.; Evangelisti M.; Gaita-Ariño A.; Sesé J.; van Slageren J.; Coronado E.; Luis F. Gd-Based Single-Ion Magnets with Tunable Magnetic Anisotropy: Molecular Design of Spin Qubits. Phys. Rev. Lett. 2012, 108, 247213 10.1103/PhysRevLett.108.247213. 23004325

[ref30] Lockyer S. J.; Chiesa A.; Brookfield A.; Timco G. A.; Whitehead G. F. S.; McInnes E. J. L.; Carretta S.; Winpenny R. E. P. Five-Spin Supramolecule for Simulating Quantum Decoherence of Bell States. J. Am. Chem. Soc. 2022, 144, 16086 10.1021/jacs.2c06384. 36007954 PMC9460766

[ref31] Chizzini M.; Crippa L.; Zaccardi L.; Macaluso E.; Carretta S.; Chiesa A.; Santini P. Quantum error correction with molecular spin qudits. Phys. Chem. Chem. Phys. 2022, 24, 20030–20039. 10.1039/d2cp01228f. 35833847

[ref32] Chizzini M.; Crippa L.; Zaccardi L.; Macaluso E.; Carretta S.; Chiesa A.; Santini P. Quantum error correction with molecular spin qudits (July, 10.1039/D2CP01228F, 2022). Phys. Chem. Chem. Phys. 2022, 24, 20565 10.1039/d2cp90132c. 35904055

[ref33] Chiesa A.; Petiziol F.; Chizzini M.; Santini P.; Carretta S. Theoretical Design of Optimal Molecular Qudits for Quantum Error Correction. J. Phys. Chem. Lett. 2022, 13, 6468–6474. 10.1021/acs.jpclett.2c01602. 35816705 PMC9310095

[ref34] Petiziol F.; Chiesa A.; Wimberger S.; Santini P.; Carretta S. Counteracting dephasing in Molecular Nanomagnets by optimized qudit encodings. npj Quantum Information 2021, 7, 133 10.1038/s41534-021-00466-3.

[ref35] Goldfarb D.; Stoll S. EPR Spectroscopy: Fundamentals and Methods; Wiley, 2018.

[ref36] Takahashi S.; Tupitsyn I. S.; van Tol J.; Beedle C. C.; Hendrickson D. N.; Stamp P. C. E. Decoherence in crystals of quantum molecular magnets. Nature 2011, 476, 76–79. 10.1038/nature10314. 21775988

[ref37] Shiddiq M.; Komijani D.; Duan Y.; Gaita-Ariño A.; Coronado E.; Hill S. Enhancing coherence in molecular spin qubits via atomic clock transitions. Nature 2016, 531, 348–351. 10.1038/nature16984. 26983539

[ref38] Aromí G.; Aguilà D.; Gamez P.; Luis F.; Roubeau O. Design of magnetic coordination complexes for quantum computing. Chem. Soc. Rev. 2012, 41, 537–546. 10.1039/c1cs15115k. 21818467

[ref39] Nielsen M. A.; Chuang I. L. Quantum Computation and Quantum Information; Cambridge University Press, 2000.

[ref40] Aguilà D.; Barrios L. A.; Velasco V.; Roubeau O.; Repollés A.; Alonso P. J.; Sesé J.; Teat S. J.; Luis F.; Aromí G. Heterodimetallic [LnLn’] Lanthanide Complexes: Toward a Chemical Design of Two-Qubit Molecular Spin Quantum Gates. J. Am. Chem. Soc. 2014, 136, 14215–14222. 10.1021/ja507809w. 25203521 PMC4195387

[ref41] Luis F.; Repollés A.; Martínez-Pérez M. J.; Aguilà D.; Roubeau O.; Zueco D.; Alonso P. J.; Evangelisti M.; Camón A.; Sesé J.; Barrios L. A.; Aromí G. “Molecular Prototypes for Spin-Based CNOT and SWAP Quantum Gates”. Phys. Rev. Lett. 2011, 107, 117203–117205. 10.1103/physrevlett.107.117203. 22026699

[ref42] Pedersen K. S.; Dreiser J.; Weihe H.; Sibille R.; Johannesen H. V.; Sørensen M. A.; Nielsen B. E.; Sigrist M.; Mutka H.; Rols S.; Bendix J.; Piligkos S. Design of Single-Molecule Magnets: Insufficiency of the Anisotropy Barrier as the Sole Criterion. Inorg. Chem. 2015, 54, 7600–7606. 10.1021/acs.inorgchem.5b01209. 26201004

[ref43] Timco G. A.; Carretta S.; Troiani F.; Tuna F.; Pritchard R. J.; Muryn C. A.; McInnes E. J. L.; Ghirri A.; Candini A.; Santini P.; Amoretti G.; Affronte M.; Winpenny R. E. P. Engineering the coupling between molecular spin qubits by coordination chemistry. Nat. Nanotechnol. 2009, 4, 173–178. 10.1038/nnano.2008.404. 19265847

[ref44] Timco G. A.; Faust T. B.; Tuna F.; Winpenny R. E. P. Linking heterometallic rings for quantum information processing and amusement. Chem. Soc. Rev. 2011, 40, 3067–3075. 10.1039/c0cs00151a. 21243130

[ref45] Chiesa A.; Whitehead G. F. S.; Carretta S.; Carthy L.; Timco G. A.; Teat S. J.; Amoretti G.; Pavarini E.; Winpenny R. E. P.; Santini P. Molecular nanomagnets with switchable coupling for quantum simulation. Sci. Rep. 2014, 4, 7423 10.1038/srep07423. 25502419 PMC4262827

[ref46] Ardavan A.; et al. Engineering coherent interactions in molecular nanomagnet dimers. npj Quantum Information 2015, 1, 15012 10.1038/npjqi.2015.12.

[ref47] Ferrando-Soria J.; Moreno Pineda E.; Chiesa A.; Fernandez A.; Magee S. A.; Carretta S.; Santini P.; Vitorica-Yrezabal I. J.; Tuna F.; Timco G. A.; McInnes E. J. L.; Winpenny R. E. P. A modular design of molecular qubits to implement universal quantum gates. Nat. Commun. 2016, 7, 11377 10.1038/ncomms11377. 27109358 PMC4848482

[ref48] Collett C. A.; Santini P.; Carretta S.; Friedman J. R. Constructing clock-transition-based two-qubit gates from dimers of molecular nanomagnets. Phys. Rev. Res. 2020, 2, 032037 10.1103/physrevresearch.2.032037.

[ref49] Giannoulis A.; Ben-Ishay Y.; Goldfarb D. Methods Enzymol.; Cotruvo J. A., Ed.; Academic Press, 2021; Vol. 651, pp 235–290. 10.1016/bs.mie.2021.01.040 33888206

[ref50] Raitsimring A. M.; Gunanathan C.; Potapov A.; Efremenko I.; Martin J. M. L.; Milstein D.; Goldfarb D. Gd3+ Complexes as Potential Spin Labels for High Field Pulsed EPR Distance Measurements. J. Am. Chem. Soc. 2007, 129, 14138–14139. 10.1021/ja075544g. 17963387

[ref51] EL Mkami H.; Hunter R. I.; Cruickshank P. A. S.; Taylor M. J.; Lovett J. E.; Feintuch A.; Qi M.; Godt A.; Smith G. M. High-sensitivity Gd3+–Gd3+ EPR distance measurements that eliminate artefacts seen at short distances. Magn. Reson. 2020, 1, 301–313. 10.5194/mr-1-301-2020. PMC10500690 37904818

[ref52] Bernhardt P. V.; Flanagan B. M.; Riley M. J. Isomorphous Lanthanide Complexes of a Tripodal N4O3 Ligand. Aust. J. Chem. 2000, 53, 229 10.1071/ch99175.

[ref53] Bernhardt P. V.; Flanagan B. M.; Riley M. J. Completion of the Isomorphous Ln(trensal) Series. Aust. J. Chem. 2001, 54, 229 10.1071/ch01076.

[ref54] Habib M.; Sain S.; Das B.; Chandra S. K. Benign routes for the syntheses of polydentate Schiff base and their lanthanide complexes. J. Indian Chem. Soc. 2011, 88, 1501.

[ref55] Kanesato M.; Mizukami S.; Houjou H.; Tokuhisa H.; Koyama E.; Nagawa Y. Comparison of the bond lengths for the lanthanide complexes of tripodal heptadentate ligands. J. Alloys Compd. 2004, 374, 307–310. 10.1016/j.jallcom.2003.11.096.

[ref56] Kanesato M.; Yokoyama T. Synthesis and Structural Characterization of Ln(III) Complexes (Ln = Eu, Gd, Tb, Er, Tm, Lu) of Tripodal Tris[2-(salicylideneamino)ethyl]amine. Chem. Lett. 1999, 28, 137–138. 10.1246/cl.1999.137.

[ref57] Kanesato M.; Yokoyama T. Crystal Structures of Dysprosium(III) and Holmium(III) Complexes of Tripodal Tris[2-(salicylideneamino)ethyl]amine. Anal. Sci. 2000, 16, 335–336. 10.2116/analsci.16.335.

[ref58] Kanesato M.; Yokoyama T.; Itabashi O.; Suzuki T. M.; Shiro M. Synthesis and Structural Characterization of Praseodymium(III) and Neodymium(III) Complexes of Tripodal Tris[2-(salicylideneamino)ethyl]amine. Bull. Chem. Soc. Jpn. 1996, 69, 1297–1302. 10.1246/bcsj.69.1297.

[ref59] Pedersen K. S.; Ungur L.; Sigrist M.; Sundt A.; Schau-Magnussen M.; Vieru V.; Mutka H.; Rols S.; Weihe H.; Waldmann O.; Chibotaru L. F.; Bendix J.; Dreiser J. Modifying the properties of 4f single-ion magnets by peripheral ligand functionalisation. Chem. Sci. 2014, 5, 1650–1660. 10.1039/c3sc53044b.

[ref60] Coffman R. E.; Buettner G. R. General magnetic dipolar interaction of spin-spin coupled molecular dimers. Application to an EPR spectrum of xanthine oxidase. J. Phys. Chem. 1979, 83, 2392–2400. 10.1021/j100481a018.

[ref61] Hahn E. L. Spin Echoes. Phys. Rev. 1950, 80, 580–594. 10.1103/physrev.80.580.

[ref63] Buch C. D.; Hansen S. H.; Mitcov D.; Tram C. M.; Nichol G. S.; Brechin E. K.; Piligkos S. Design of pure heterodinuclear lanthanoid cryptate complexes. Chem. Sci. 2021, 12, 6983–6991. 10.1039/d1sc00987g. 34123326 PMC8153240

