# Supplementary material for: Dipolar-Coupled Entangled Molecular 4f Qubits
Source: J Am Chem Soc. 2023 Jan 25;145(5):2877–83. doi: 10.1021/jacs.2c10902 (PMC9912257; doi:10.1021/jacs.2c10902)
Supplement: Supplementary file 1 — ja2c10902_si_001.pdf [file ja2c10902_si_001.pdf]

Supporting information

for

Dipolar-Coupled Entangled Molecular 4f Qubits

Bela E. Bode,[1] Edoardo Fusco,[1] Rachel Nixon[1] Christian D. Buch,[2] Høgni Weihe,[2] and Stergios Piligkos*[2]*

[1]EaStCHEM School of Chemistry, Biomedical Sciences Research Complex, and Centre of Magnetic Resonance, University of St Andrews, North Haugh, St Andrews KY16 9ST, UK

[2]Department of Chemistry, University of Copenhagen, DK-2100 Copenhagen, Denmark

Contents

Experimental....................2

Crystallographic Tables....................4

C.w.-single crystal Electron Paramagnetic Resonance....................8

Pulsed single crystal Electron Paramagnetic Resonance....................14

References....................27

Experimental

Materials and methods

Acetonitrile, tris(2-aminoethyl)amine, Yb_2O_3, Lu_2O_3 and salicylaldehyde were purchased from commercial sources and used as received. $Yb(OTf)_3·9H_2O$ and $Lu(OTf)_3·9H_2O$ were synthesized from the corresponding Ln_2O_3 following a literature procedure.[1] To determine the water content of the triflate salts an EDTA titration using xylenol orange as the indicator was performed on each triflate salt.

Inductively coupled plasma mass spectrometry (ICP-MS) was performed on a Bruker Aurora Elite at The Department of Chemistry, University of Copenhagen. Calibration solutions were made by diluting a reference solution from Inorganic Ventures in 2 % nitric acid of TraceSelect grade. Prior to use the instrument was tuned and calibrated, using calibration points spanning the range of metal ion concentrations in the samples. The sample solution was prepared by dissolving crystals (from the same batch as the single crystal of **1** was isolated from) in diluted, boiling nitric acid of TraceSelect grade. Two samples for measurement were then prepared by diluting the sample solution with 2 % nitric acid (TraceSelect grade) until the Yb and Lu concentrations were within the interval 1 – 50 ng/ml. The ICP-MS analysis gave an Yb:Lu ratio of 9.5:990.5.

Continous wave Electron Paramagnetic Resonance (c.w.-EPR) measurements at X-band frequencies were measured on a Bruker Elexsys E500 equipped with an automatic goniometer for rotations of single crystals and an Oxford Instruments cryostat operating at 5 – 300 K.

Pulse EPR measurements were performed using an X-band (9.3 GHz) Bruker ELEXSYS E 580 spectrometer combined with a cryogen-free variable temperature cryostat (Cryogenics VTU). The temperature was varied in a range of 2 to 18 K.

EDFS were performed at 11 K using a Hahn-echo $\pi/2$-τ-π-τ-echo sequence with a π pulse length of 32 ns and a $\pi/2$ of 16 ns, a τ of 300 ns, a shot repetition time of 50 μs and a microwave power attenuation adjusted to maximize the echo intensity. Phase memory time (T_m) measurements (Hahn-echo $\pi/2$-τ-π-τ-echo) were performed with a starting τ of 500 ns and pulse lengths of 128/256 ns for $\pi/2$ and π (250 ns τ and 64/128 ns for $\pi/2$ and π, respectively, for experiments above 10 K), respectively. The shot repetition time was set to 15 ms at 2 K, 5 ms at 3.5 K, 500 μs at 5 K, 200 μs at 6.5 K, 100 μs at 8 K and 170 μs at all higher temperatures (limited by amplifier duty cycle). To extract T_m the exponential decay of the echo as a function of 2τ was fitted to a monoexponential of the form $[I(\tau) = I_0 + A·\exp(-2\tau/T_m)]$, where A is a preexponential factor. The spin-lattice relaxation (T_1) was measured using an inversion recovery pulse sequence (π-T-$\pi/2$-τ-π-echo), with a 300 ns (200 ns at 14 K and above) τ , pulse lengths of 16/32 ns for $\pi/2$ and π preceded by a noncoherent 12 ns π inversion pulse, T of 1000 ns (500 ns at 14 K and above) and a time step adjusted at each temperature: 120 μs at 2 K, 30 μs at 3.5 K, 5 μs at 5 K, 2.5 μs at 6.5 K, 0.150 μs at 8 K and 11 K, 50 ns at 14 K, and 16 ns at 18 K. The shot repetition time was set to 70 ms at 2 K, 18 ms at 3.5 K, 3 ms at 5 K, 1.5 ms at 6.5 K, 300 μs at 8 K, 100 μs at 11 K, and 65 μs at all higher temperatures (limited by amplifier duty cycle). T_1 was determined by fitting a biexponential $[I(t) = I_0 + A_f·\exp(T/T_{1f}) + A_s·\exp(T/T_{1s})]$ to the data (for the temperatures 2, 3.5, 5 and 6.5 K) or a monoexponential $[I(t) = I_0 + A·\exp(T/T_1)]$ (for temperatures above 6.5 K). In the exponentials A_i is a preexponential factor. For the biexponential fits the slow relaxation (T_{1s}) was used as the T_1 of the system, as the faster process is presumed to be due to spectral diffusion. The nutation experiments

(t_p-T-π/2-τ-π-τ-echo) were performed at 8 K with a shot repetition time of 150 µs and used τ of 200 ns and T of 6000 ns, and a starting value of 2 ns t_p with a 2 ns increment. The respective pulse length of π and π/2 pulses was adjusted to the microwave power attenuation: 12 and 6 ns for 4 to 12 dB, 20 and 10 ns for 15 dB, 28 and 14 ns for 18 dB and 40 and 20 ns for 22 dB. The nutation frequencies were determined by zero-filling, Hamming windowing and Fourier transforming the time traces.

Synthesis

Single crystals of **1** were prepared analogously to a literature procedure.[2] **1** was prepared employing a molar ratio of 1:99 for $Yb(OTf)_3 \cdot 9H_2O$ and $Lu(OTf)_3 \cdot 9H_2O$, respectively.

Crystallographic Tables

Table S1. Crystallographic data and refinement parameters for Yb(trensal).[3]

Empirical formula	$C_{27}H_{27}N_4O_3Yb$
Formula weight	628.58
T/K	122(1)
Crystal system	trigonal
Space group	$P\bar{3}c1$
a/Å	12.9293(4)
b/Å	12.9293(4)
c/Å	16.3080(6)
α/°	90
β/°	90
γ/°	120
V/Å^3	2360.92(13)
Z	4
ρ_{calc} / g cm^{-3}	1.7683
μ/mm^{-1}	3.999
F(000)	1244
Crystal size/mm^3	0.5 × 0.5 × 0.5
Radiation	Mo Kα (λ = 0.71073)
2θ range for data collection/°	5 to 61.98
Index ranges	$-18 \leq h \leq 18$, $-16 \leq k \leq 17$, $-20 \leq l \leq 23$
Reflections collected	19581
Independent reflections	2515 [R_{int} = 0.0458, R_{sigma} = 0.0292]
Data/restraints/parameters	2515/0/106
Goodness-of-fit on F^2	1.040
Final R indexes [$I \geq 2\sigma(I)$]	R_1 = 0.0260, wR_2 = 0.0684
Final R indexes [all data]	R_1 = 0.0441, wR_2 = 0.0787
Largest diff. peak/hole / e Å^{-3}	2.32/-2.00

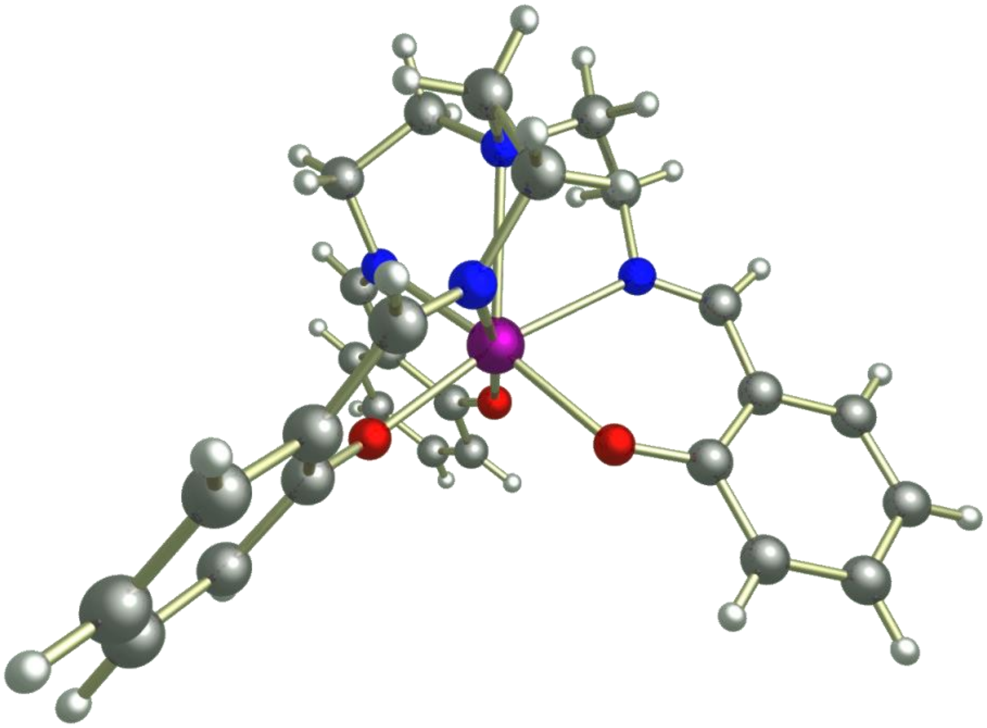

Figure S1 Molecular structure of Yb(trensal). Color code: H, white; C, grey; O, red; N, blue and Yb, purple.

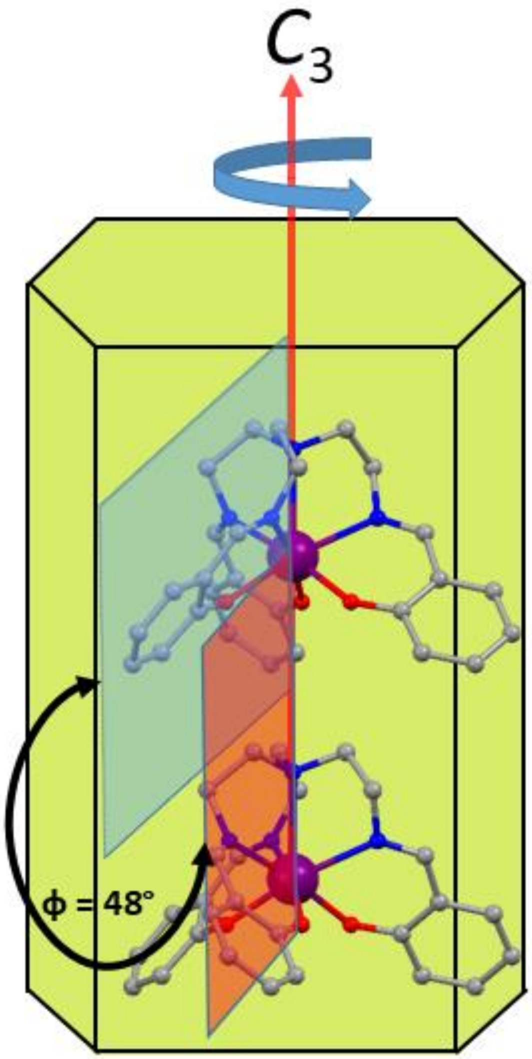

Figure S2 Orientation of molecular C_3 axis in the crystal. The two planes indicate the 48° angle between the two magnetic nonequivalent sites in the unit cell. Hydrogens have been omitted for clarity. Color code: C, grey; O, red; N, blue and Yb, purple.

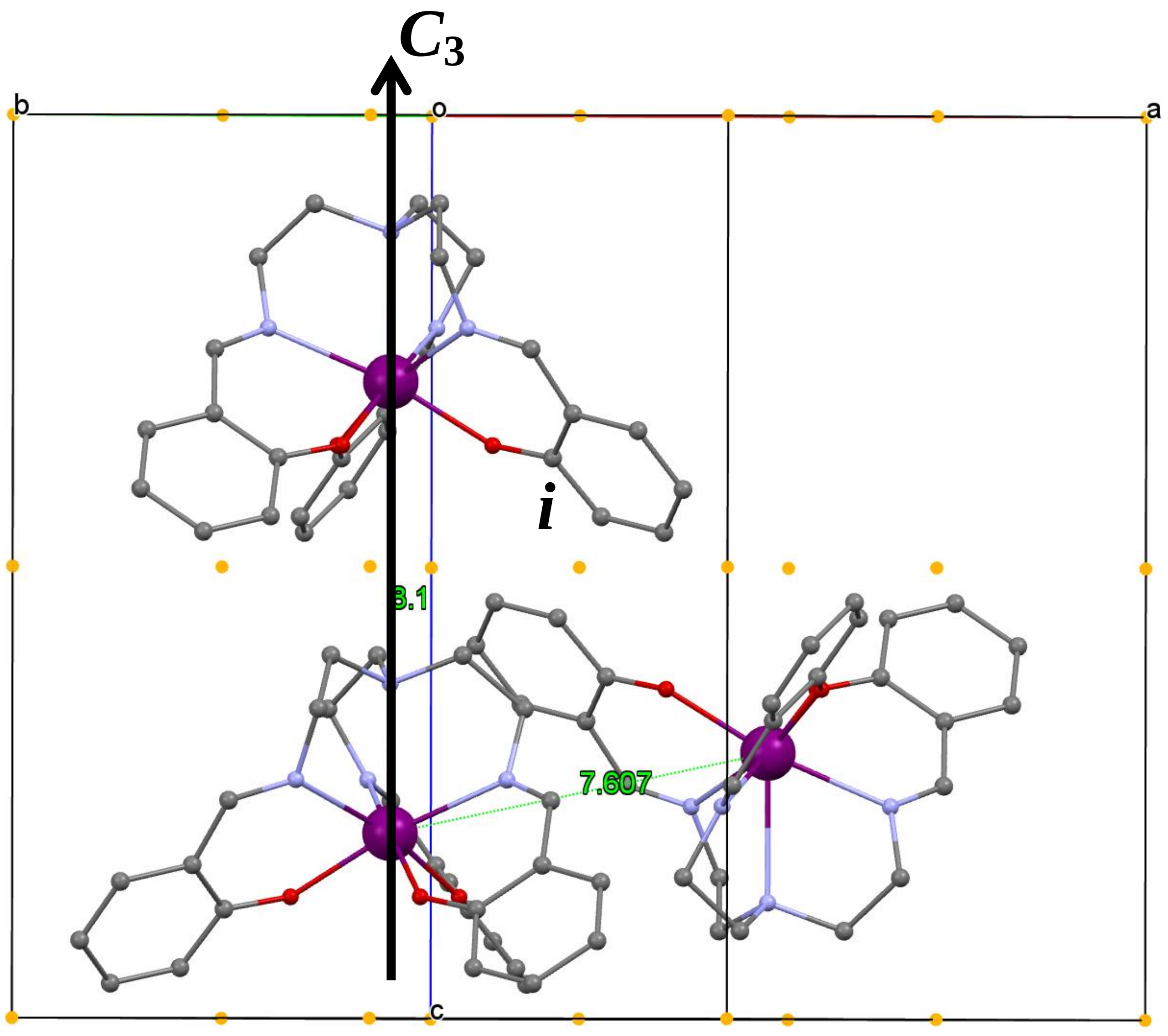

Figure S3 Unit cell of Yb(trensal) displaying three of the four crystallographically equivalent complexes in the unit cell of **1**. The numbers in green are distances in Å between neighboring Yb^{III} centers. The yellow dots indicate crystallographic inversion centers. Color code: C, grey; O, red; N, blue and Yb, purple.

C.w.-single crystal Electron Paramagnetic Resonance

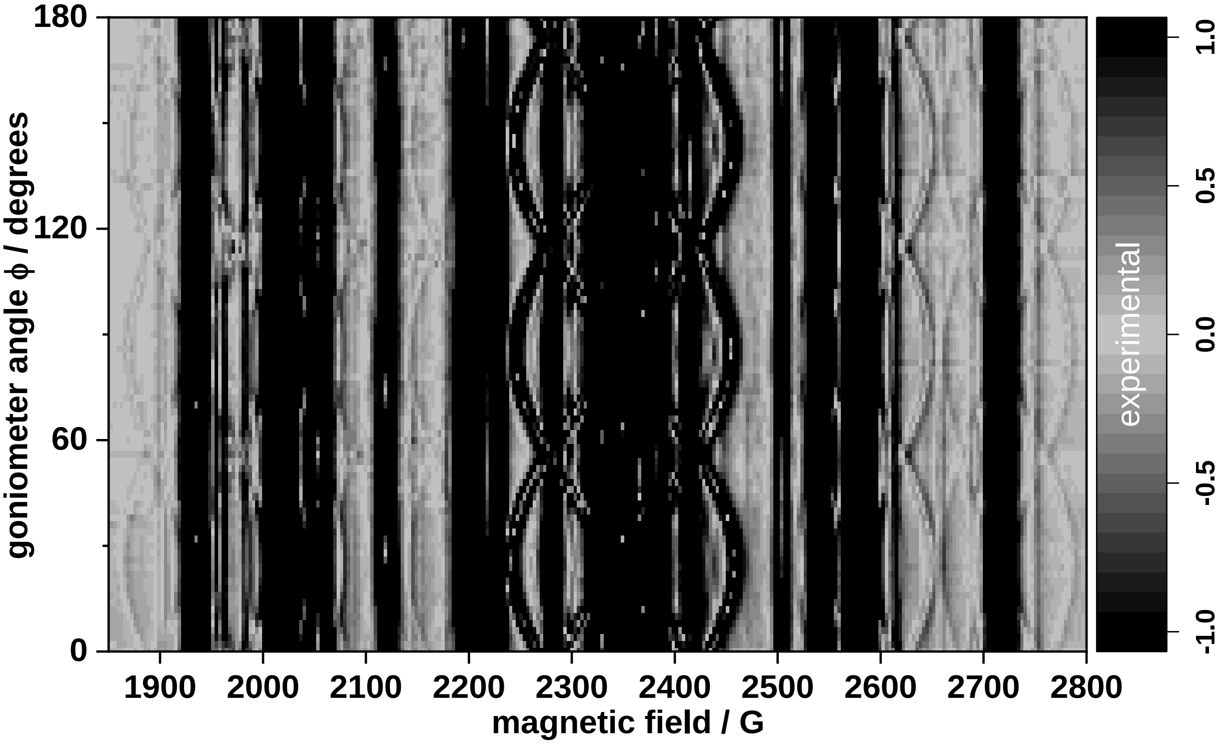

Figure S4. Angular variation of the X-band c.w.-EPR spectrum of **1** in the plane perpendicular to the C_3 axis and at 15 K.

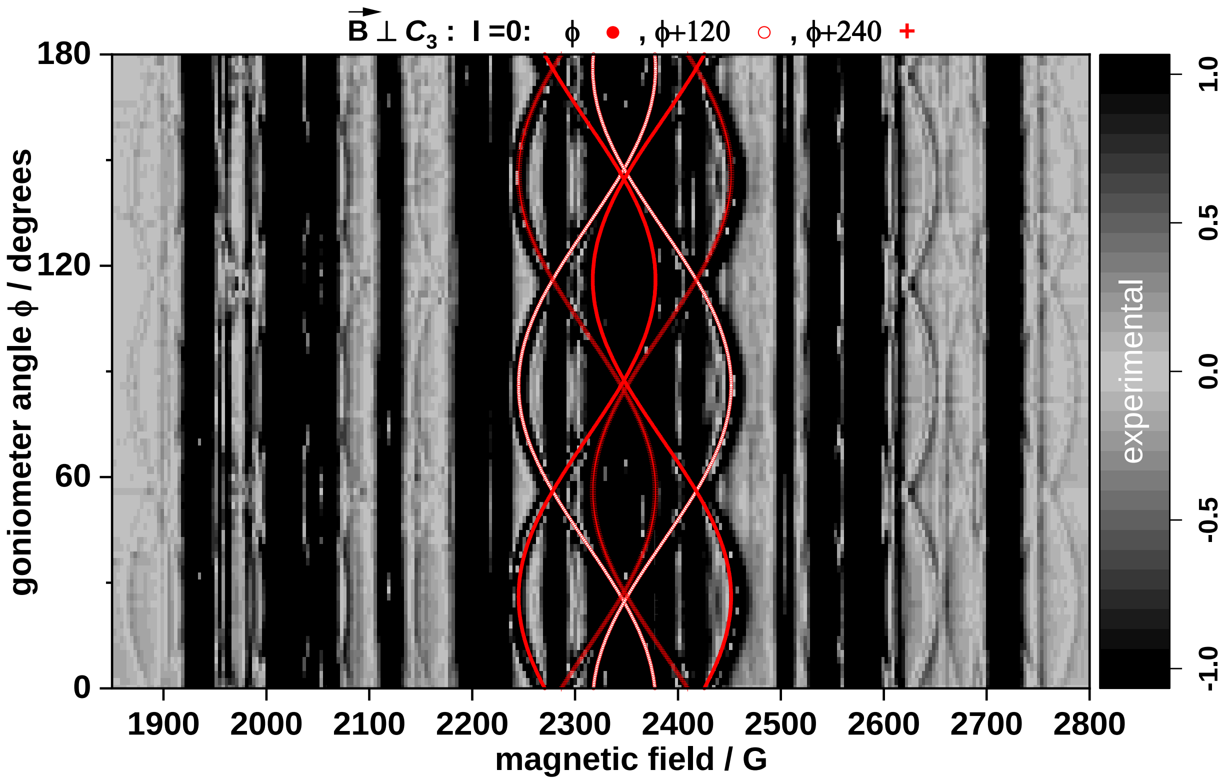

Figure S5. Angular variation of the X-band c.w.-EPR spectrum of **1** in the plane perpendicular to the C_3 axis, the internuclear Yb^{III}-Yb^{III} distance vector $\vec{R}$ at an angle θ = 78.8° to the C_3 axis, and at 15 K. φ, φ + 120 and φ + 240 indicate the three neighboring Yb(trensal) complexes at an angle θ = 78.8° to the C_3 axis (Figure 1 in the main text). Experiment in shades of gray and simulation involving the dipolar couplings between coupled Yb sites for two I = 0 isotopes (red).

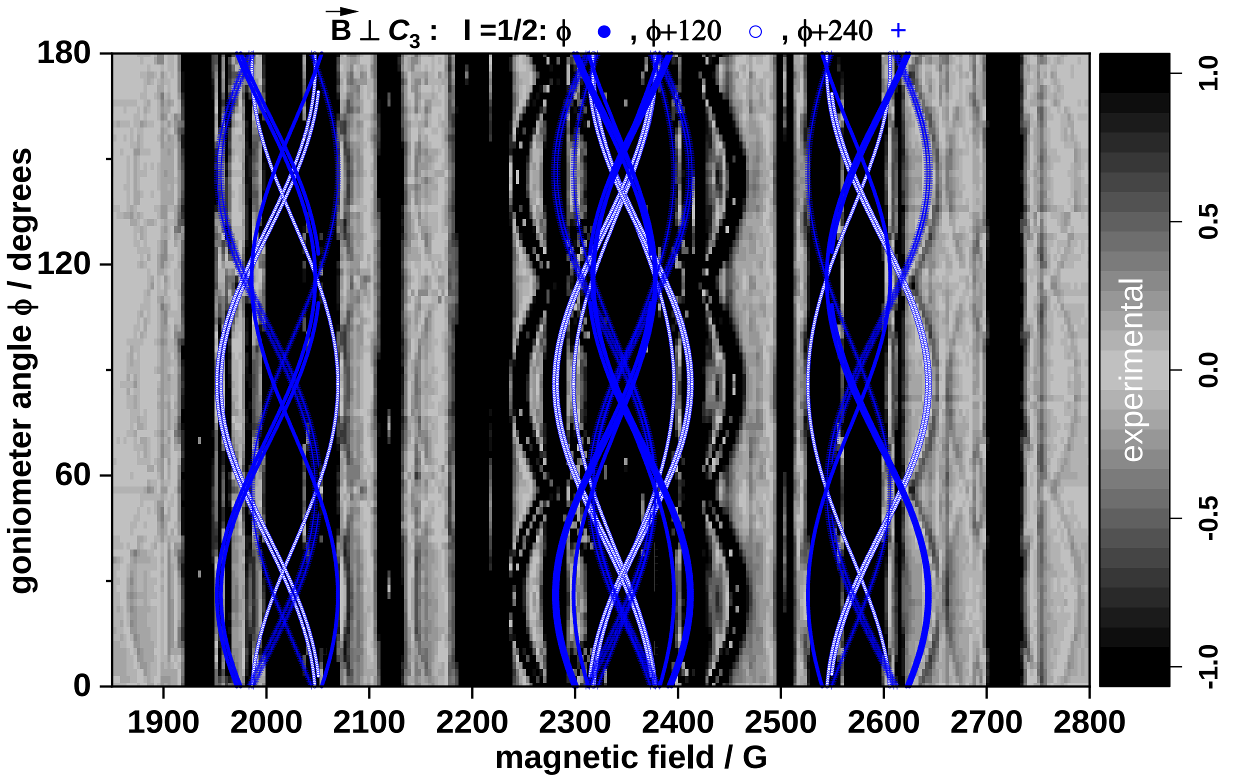

Figure S6. Angular variation of the X-band c.w.-EPR spectrum of **1** in the plane perpendicular to the C_3 axis, the internuclear Yb^{III}-Yb^{III} distance vector $\vec{R}$ at an angle θ = 78.8° to the C_3 axis, and at 15 K. φ, φ + 120 and φ + 240 indicate the three neighboring Yb(trensal) complexes at an angle θ = 78.8° to the C_3 axis (Figure 1 in the main text). Experiment in shades of gray and simulation involving the dipolar couplings between coupled Yb sites for I = 0 and I = 1/2 isotopes (blue).

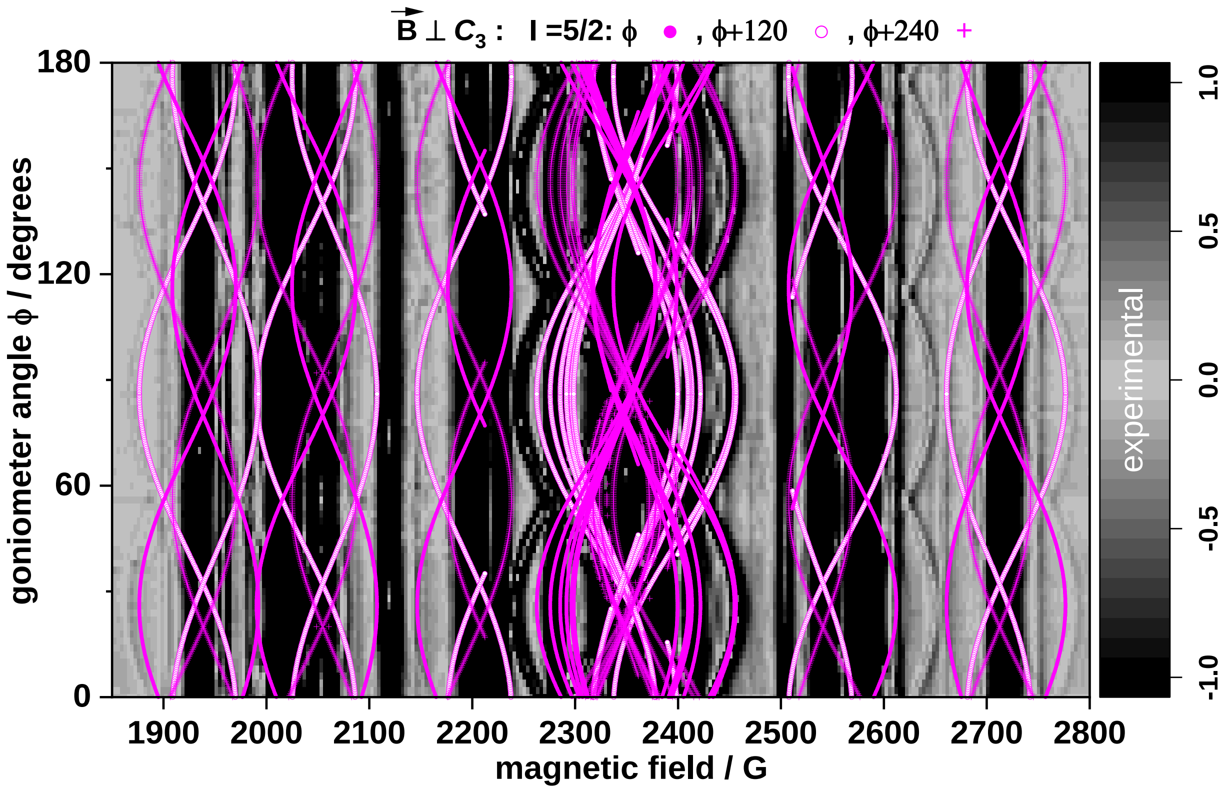

Figure S7. Angular variation of the X-band c.w.-EPR spectrum of **1** in the plane perpendicular to the C_3 axis, the internuclear Yb^{III}-Yb^{III} distance vector $\vec{R}$ at an angle θ = 78.8° to the C_3 axis, and at 15 K. φ, φ + 120 and φ + 240 indicate the three neighboring Yb(trensal) complexes at an angle θ = 78.8° to the C_3 axis (Figure 1 in the main text). Experiment in shades of gray and simulation involving the dipolar couplings between coupled Yb sites for I = 0 and I = 5/2 isotopes (purple).

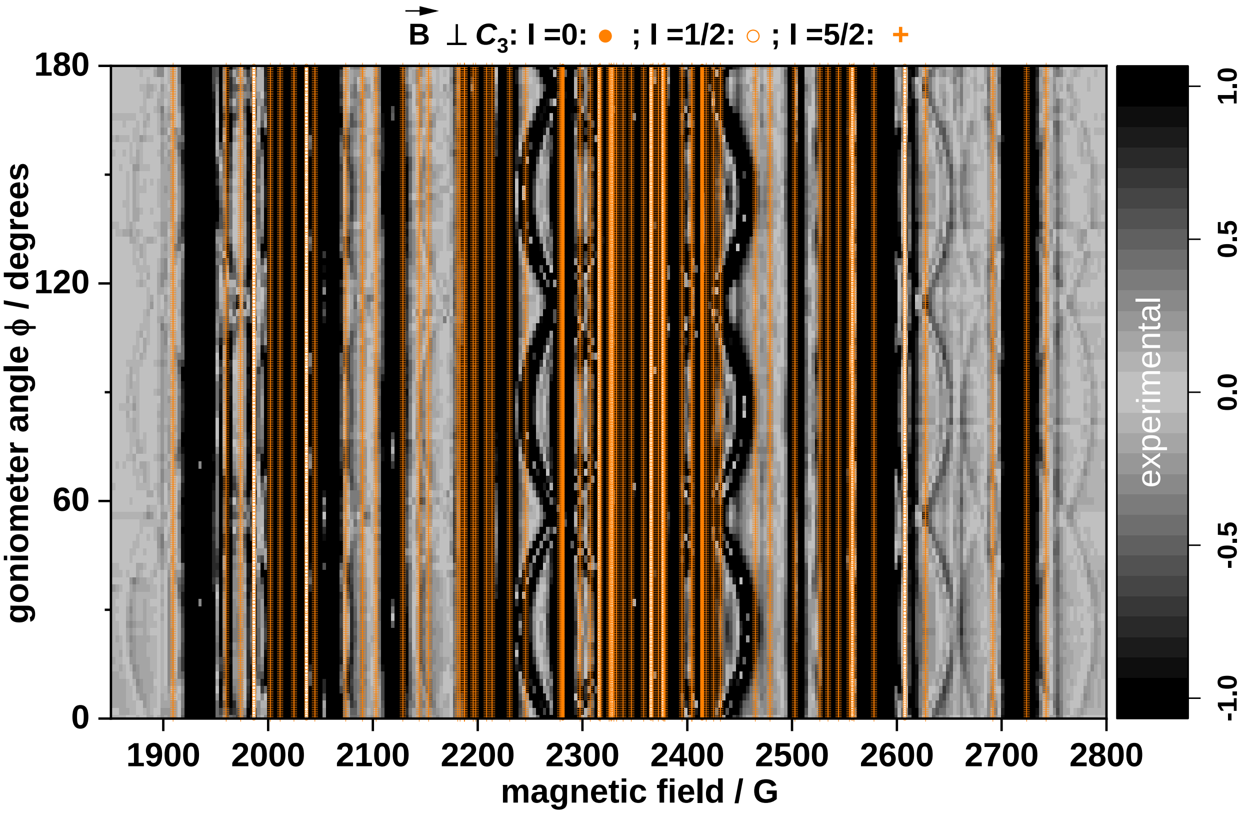

Figure S8. Angular variation of the X-band c.w.-EPR spectrum of **1** for the magnetic field perpendicular to the C_3 axis, the internuclear Yb^{III}-Yb^{III} distance vector $\vec{R}$ parallel to the C_3 axis ($\theta = 0°$), and at 15 K. Experiment in shades of gray and simulation involving the dipolar couplings between coupled Yb sites with $I = 0$ and $I = 0$, ½, 5/2 isotopes in orange.

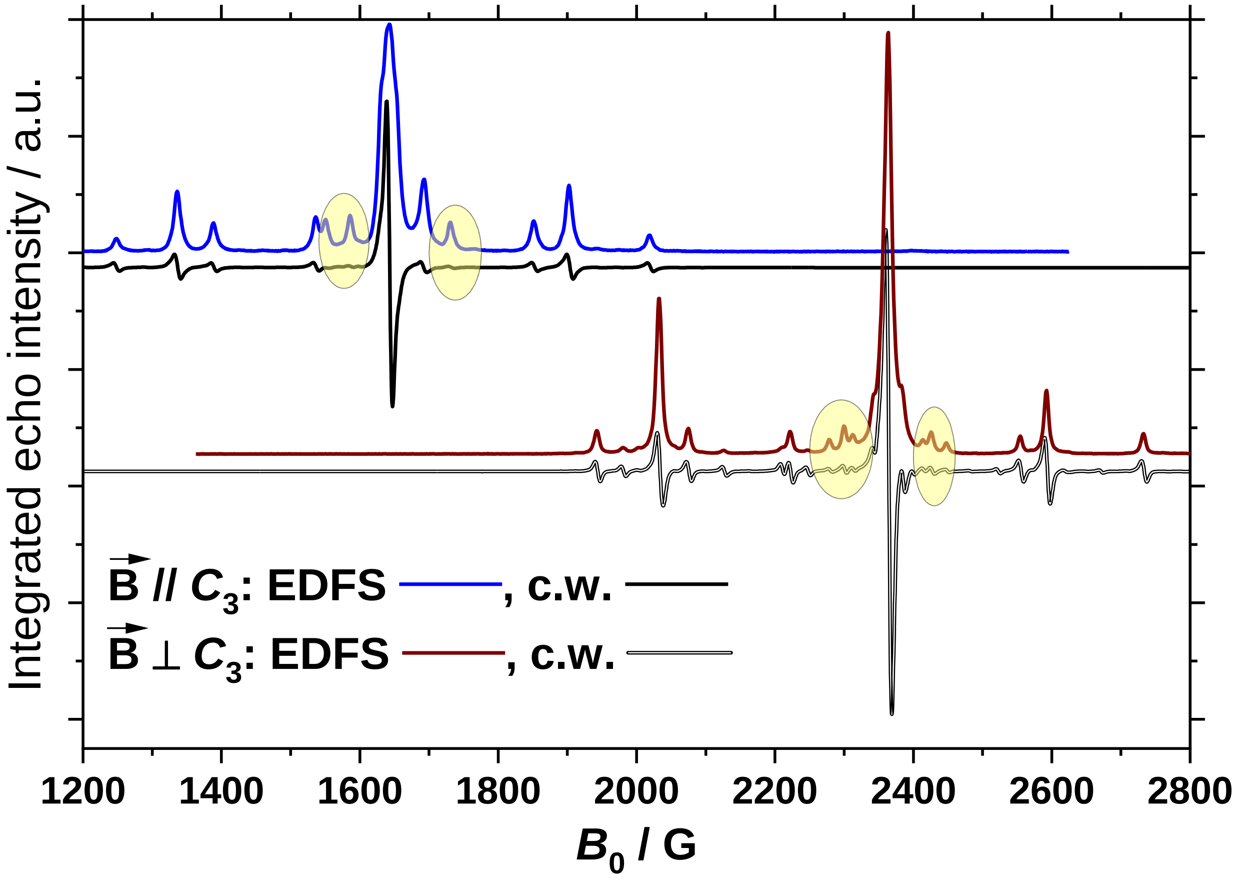

Figure S9. Comparison of EDFS and c.w.-EPR spectra of **1** with $\vec{B} \parallel C_3$ (top) and $\vec{B} \perp C_3$ (bottom). The yellow circles highlight the signals arising from dipolar interactions between neighboring Yb^{III} ions in **1**.

Pulsed single crystal Electron Paramagnetic Resonance

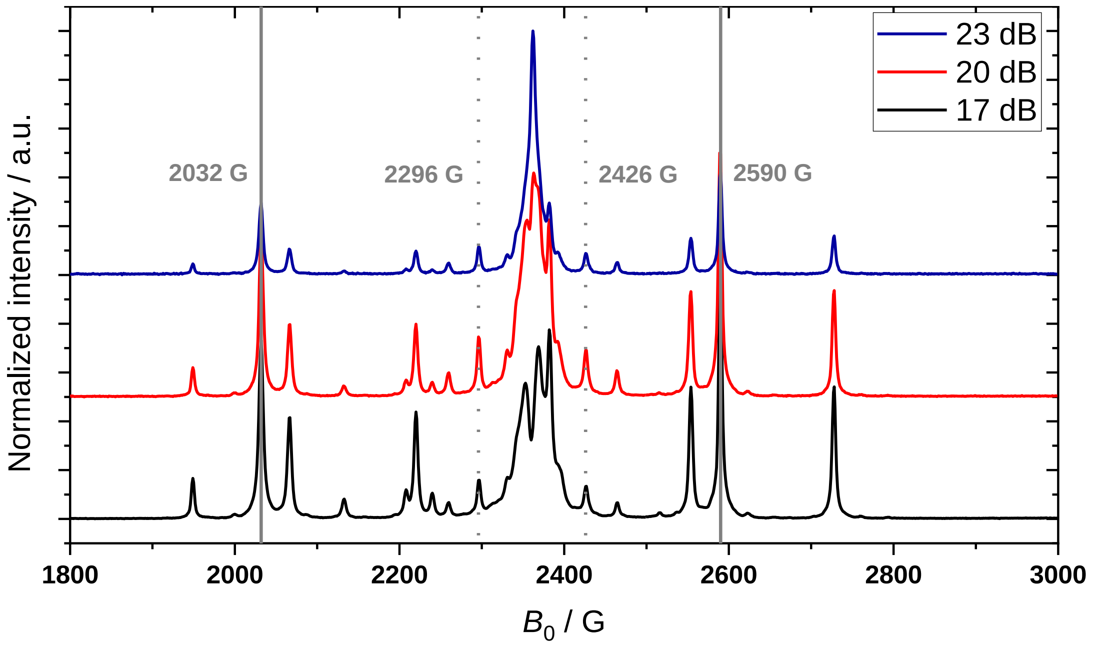

Figure S10 EDFS at different power levels measured on a single crystal of **1** oriented with $\vec{B}$ in the plane normal to the C_3 axis at 11 K. Solid lines indicate selected signals arising from isolated Yb^{III} ions, while dashed lines indicate selected signals arising from coupling between neighbouring Yb^{III} centers.

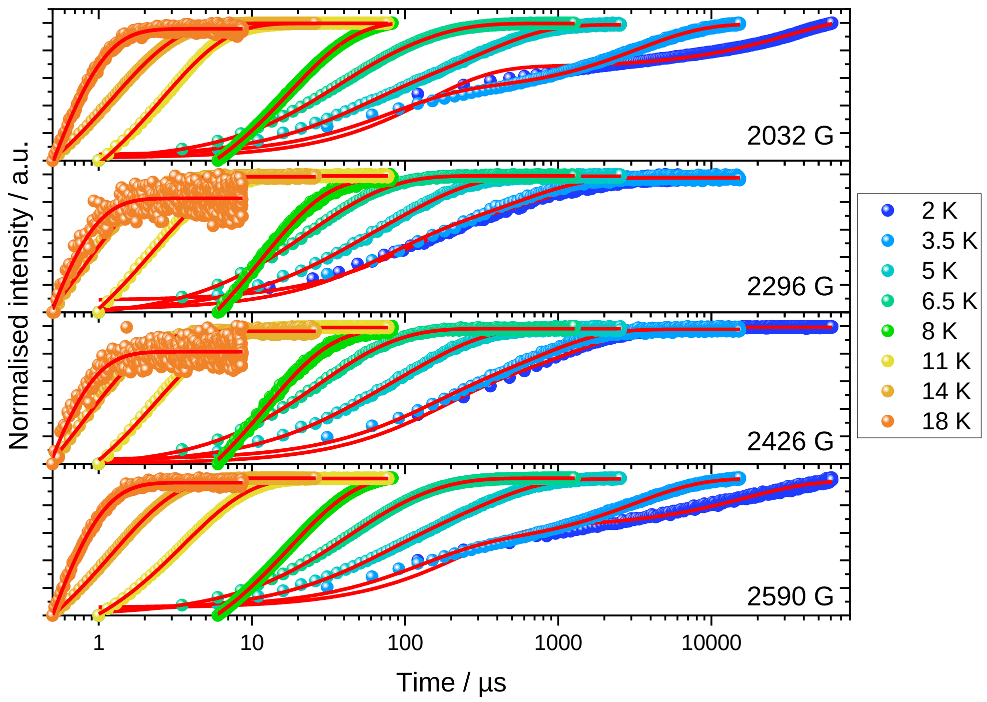

Figure S11 Normalized echo intensities (scatter) as a function of time in a standard inversion recovery sequence performed on a single crystal of **1** with $\vec{B} \perp C_3$ at selected temperatures and magnetic field positions as indicated. The red lines are the best fit to a biexponential or monoexponential function as described in the methods section.

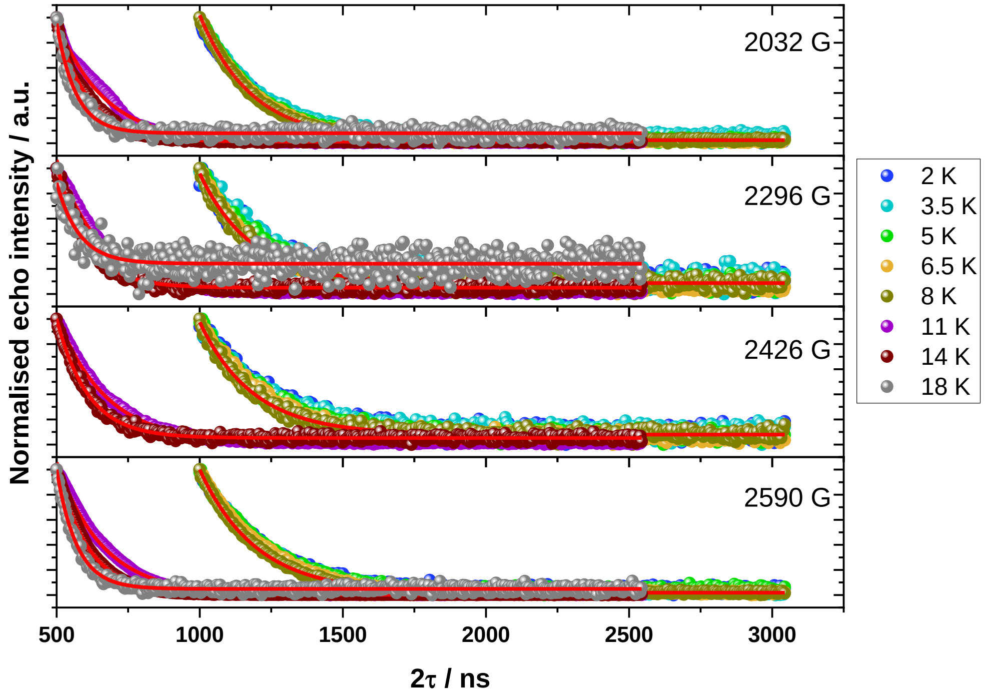

Figure S12 Normalized Hahn echo intensities (scatter) as a function of 2τ performed on a single crystal of **1** with $\vec{B} \perp C_3$ at selected temperatures and magnetic field positions as indicated. The red lines are the best fit to a monoexponential function as described in the methods section.

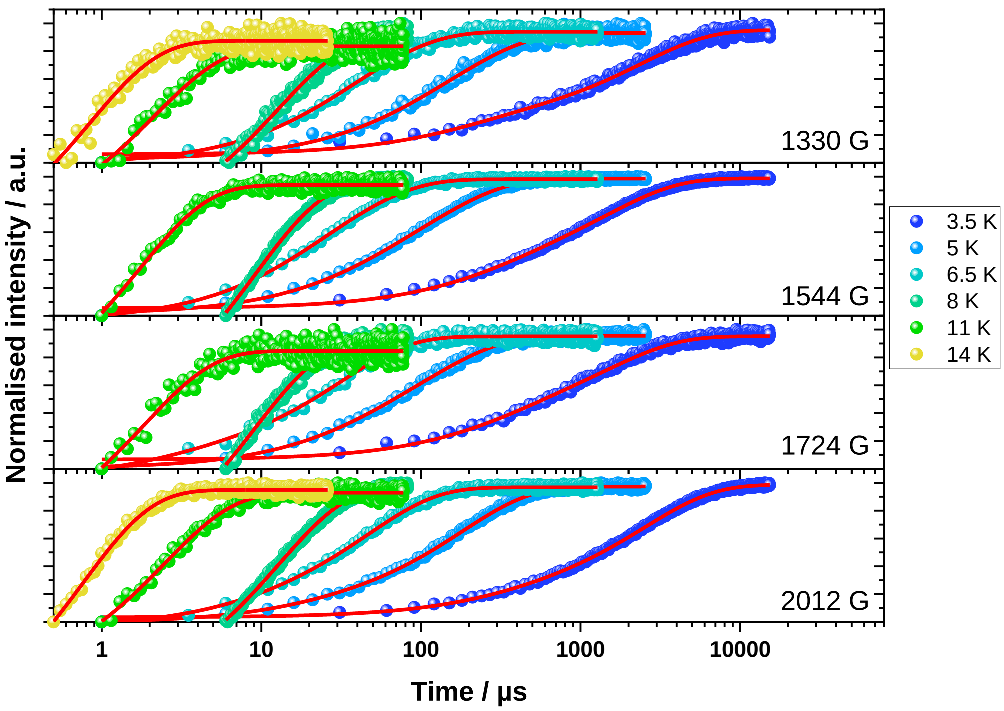

Figure S13 Normalized echo intensities (scatter) as a function of time in a standard inversion recovery sequence performed on a single crystal of **1** with $\vec{B} \parallel C_3$ at selected temperatures and magnetic field positions as indicated. The red lines are the best fit to a biexponential or monoexponential function as described in the methods section.

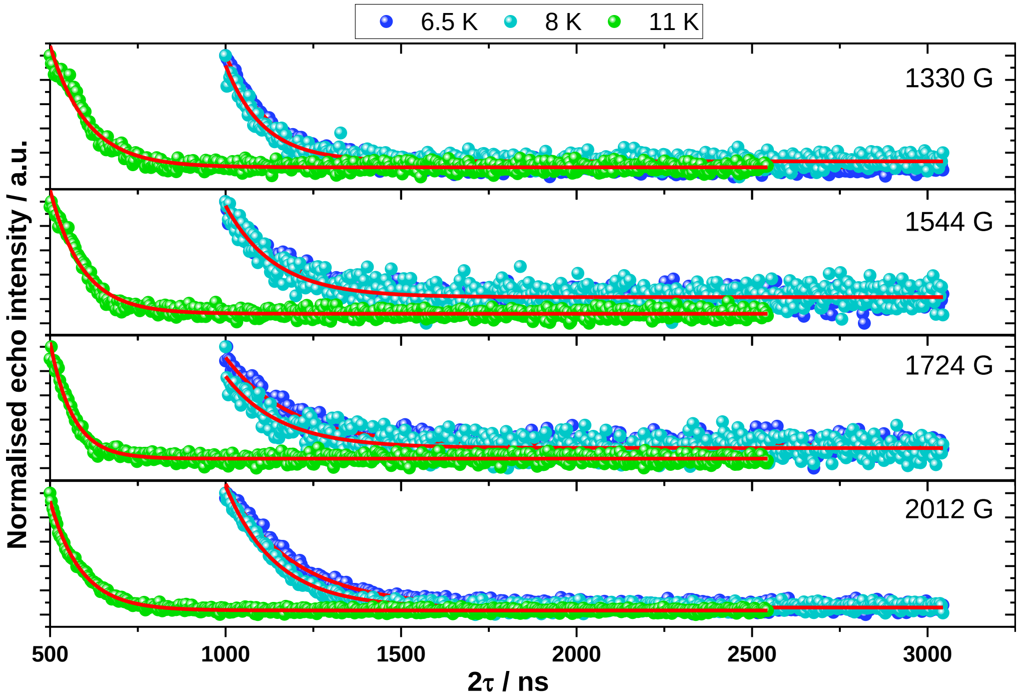

Figure S14 Normalized Hahn echo intensities (scatter) as a function of 2τ performed on a single crystal of **1** with $\vec{B} \parallel C_3$ at selected temperatures and magnetic field positions as indicated. The red lines are the best fit to a monoexponential function as described in the methods section.

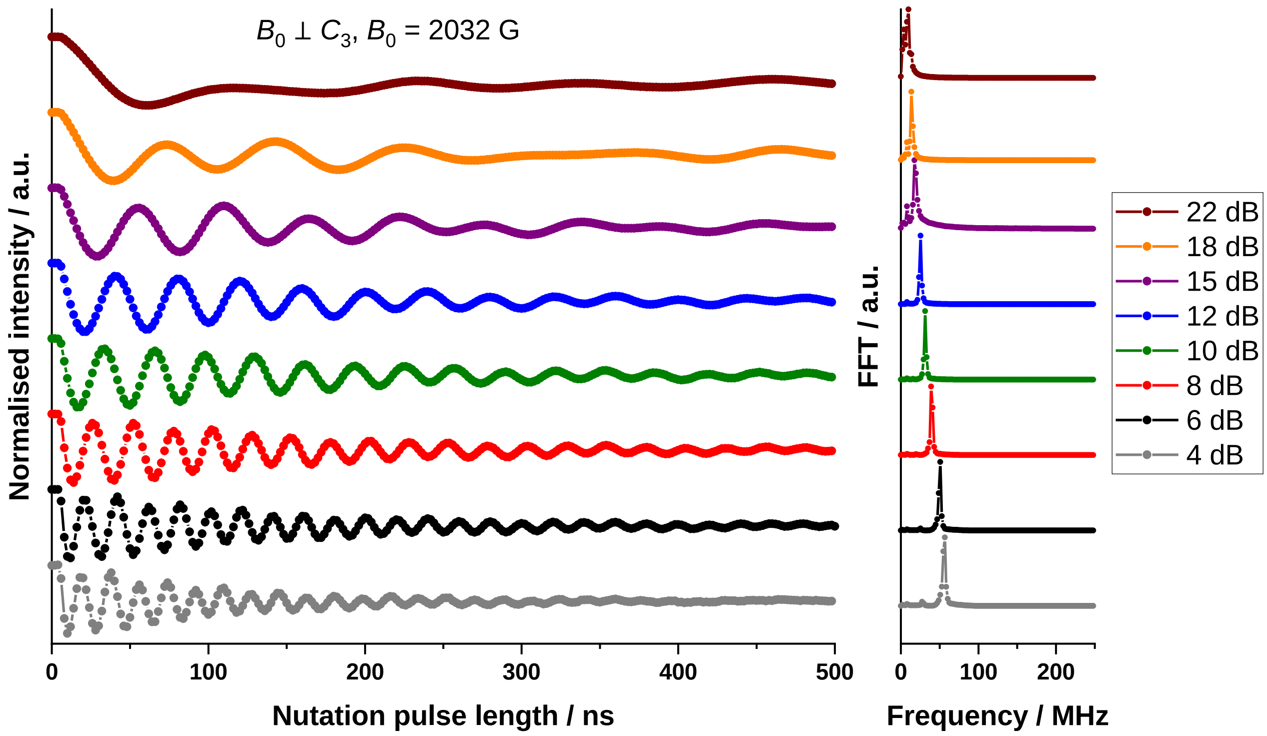

Figure S15 *Left*: Rabi oscillations as a function of microwave power for a crystal of **1** oriented with $B_0 \perp C_3$ and with B_0 = 2032 G. *Right*: Fourier transform of the Rabi oscillations. At 6dB 7 ns correspond to a π-pulse. At 4 dB the Rabi-oscillations might be undersampled. The measurements were performed at 8 K.

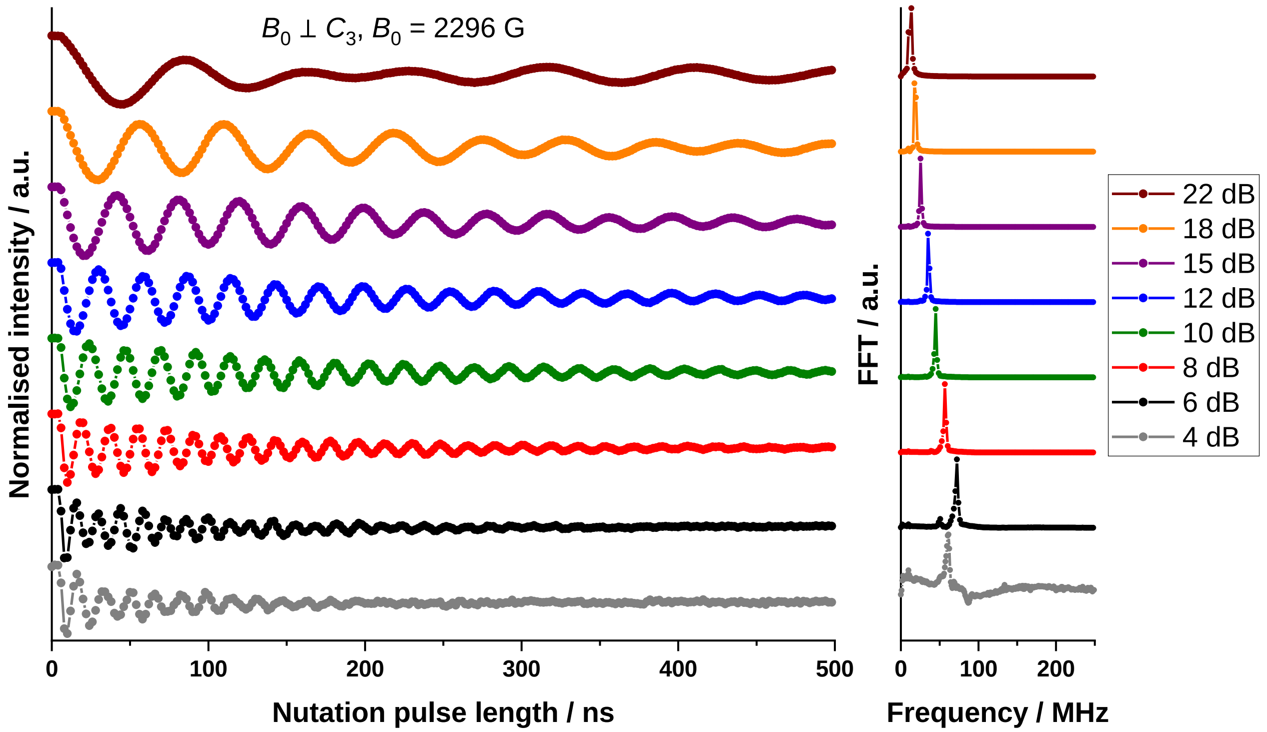

Figure S16 *Left*: Rabi oscillations as a function of microwave power for a crystal of **1** oriented with $B_0 \perp C_3$ and with B_0 = 2296 G. *Right*: Fourier transform of the Rabi oscillations. At 6dB 7 ns correspond to a π-pulse. At 4 dB Rabi-oscillations might be undersampled. The measurements were performed at 8 K.

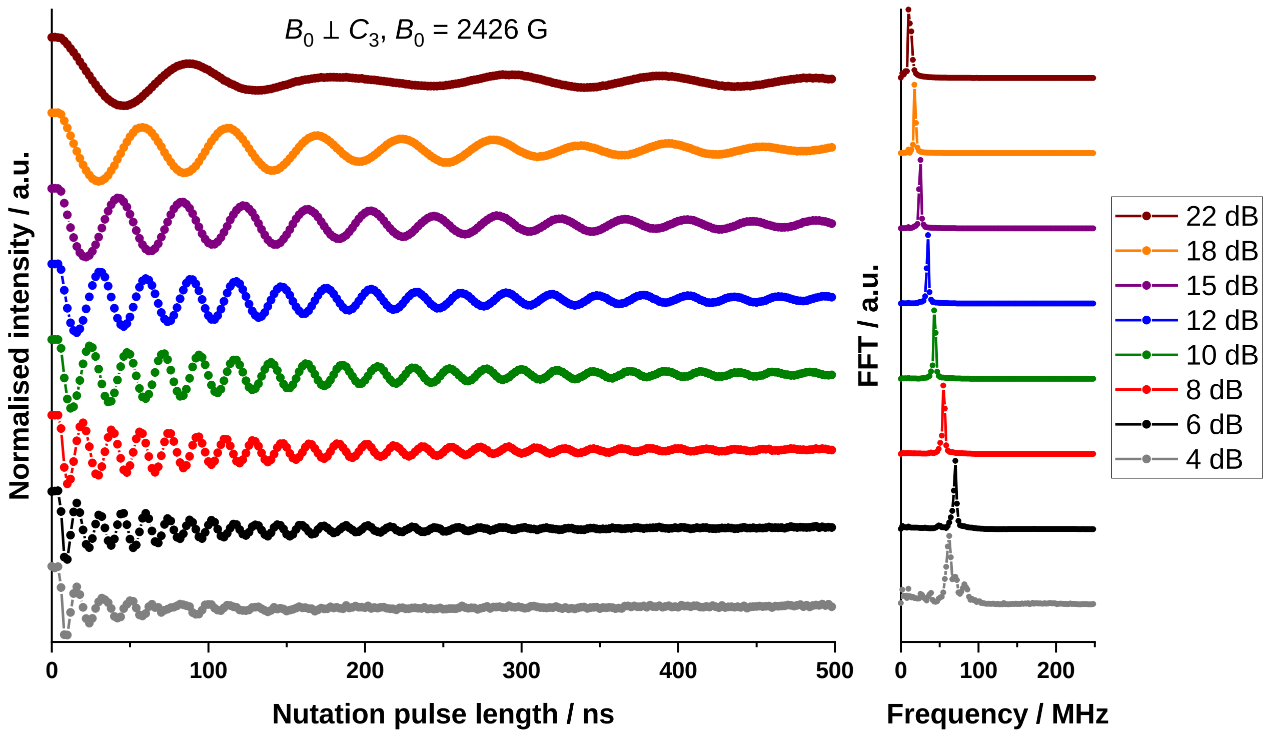

Figure S17 *Left*: Rabi oscillations as a function of microwave power for a crystal of **1** oriented with $B_0 \perp C_3$ and with B_0 = 2426 G. *Right*: Fourier transform of the Rabi oscillations. At 6dB 7 ns correspond to a π-pulse. At 4 dB the Rabi-oscillations might be undersampled. The measurements were performed at 8 K.

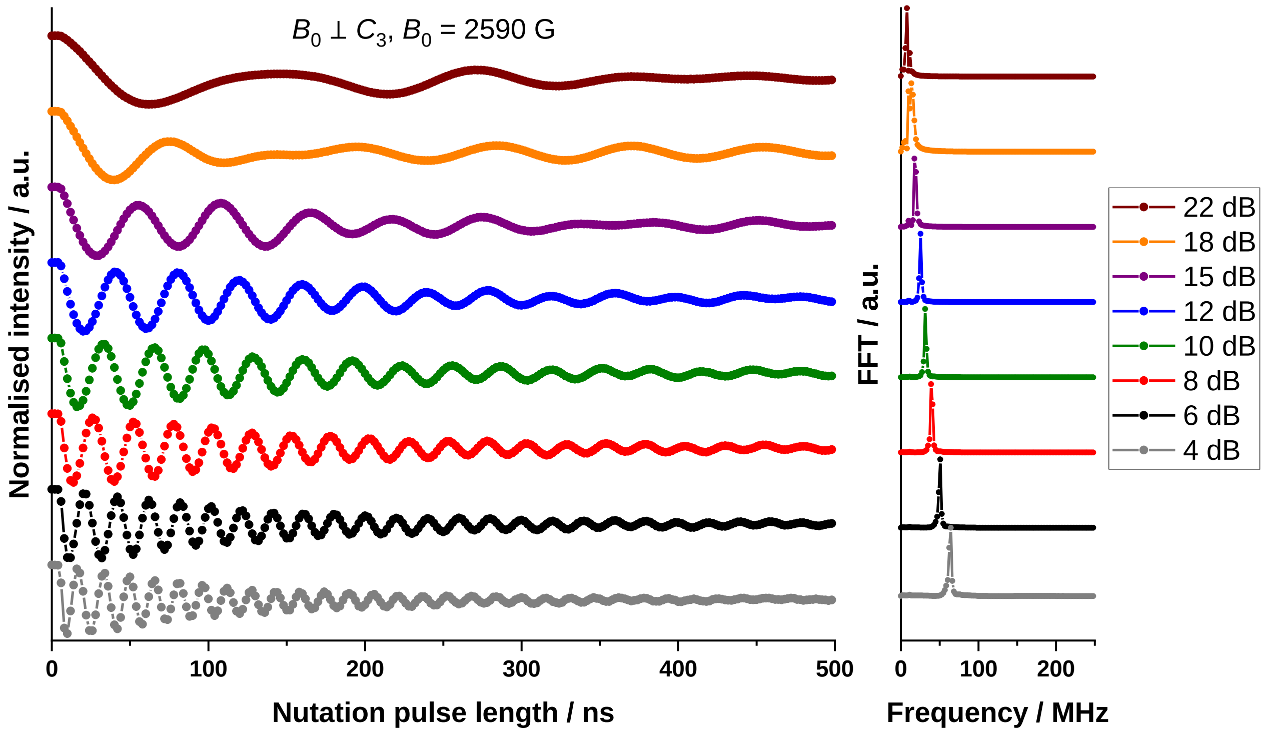

Figure S18 *Left*: Rabi oscillations as a function of microwave power for a crystal of **1** oriented with $B_0 \perp C_3$ and with B_0 = 2590 G. *Right*: Fourier transform of the Rabi oscillations. At 6dB 7 ns correspond to a π-pulse. At 4 dB the Rabi-oscillations might be undersampled. The measurements were performed at 8 K.

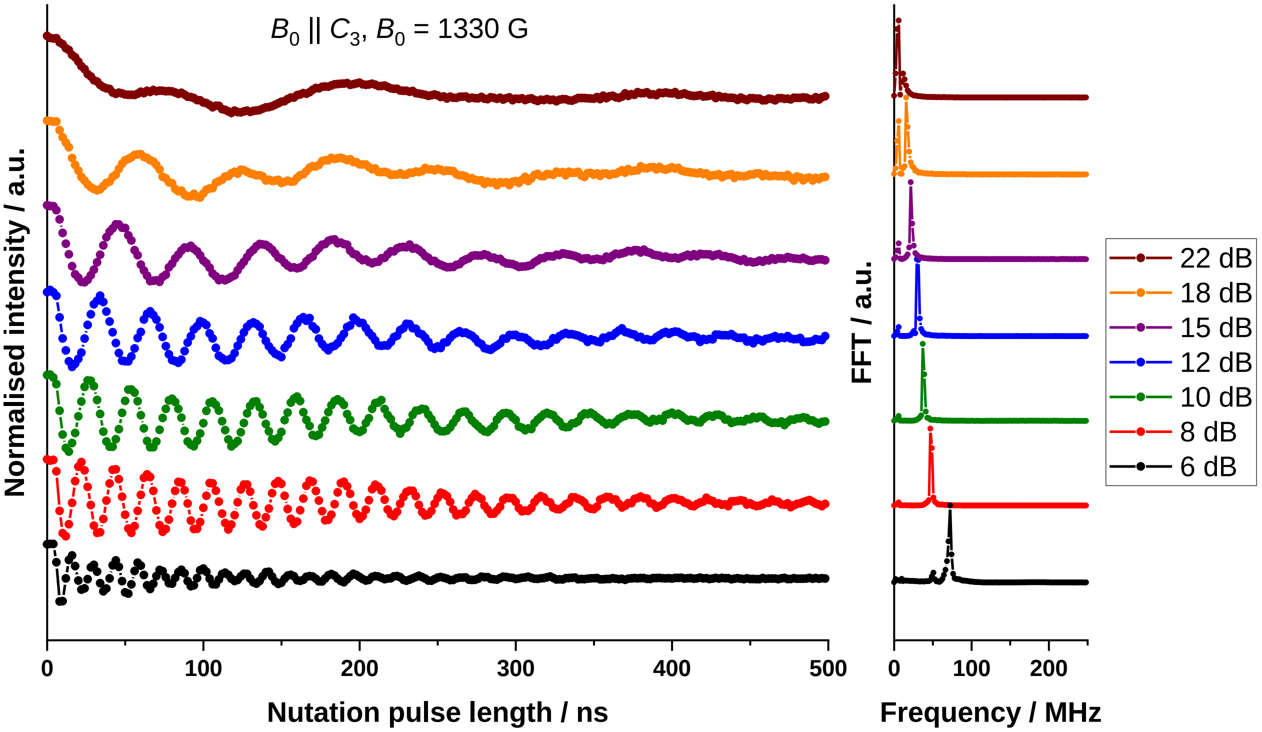

Figure S19 *Left*: Rabi oscillations as a function of microwave power for a crystal of **1** oriented with $B_0 \parallel C_3$ and with B_0 = 1330 G. *Right*: Fourier transform of the Rabi oscillations. The measurements were performed at 8 K.

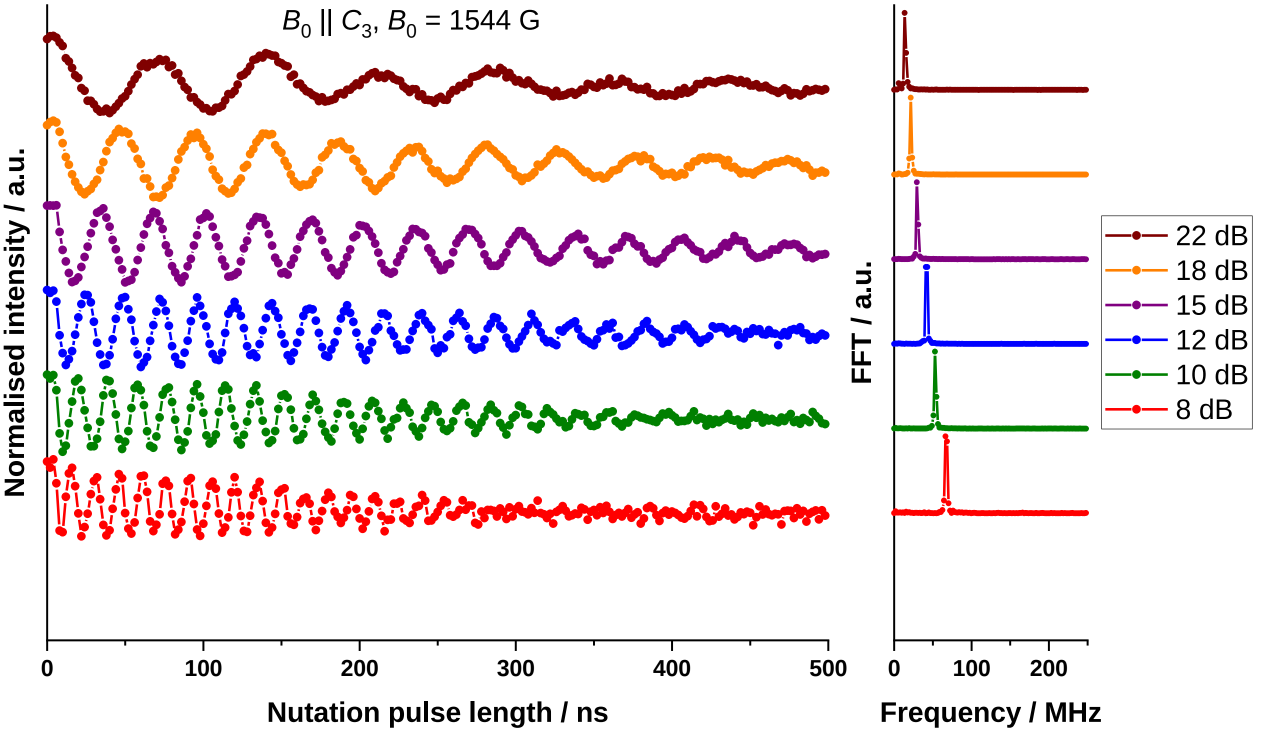

Figure S20 *Left*: Rabi oscillations as a function of microwave power for a crystal of **1** oriented with $B_0 \parallel C_3$ and with B_0 = 1544 G. *Right*: Fourier transform of the Rabi oscillations. The measurements were performed at 8 K.

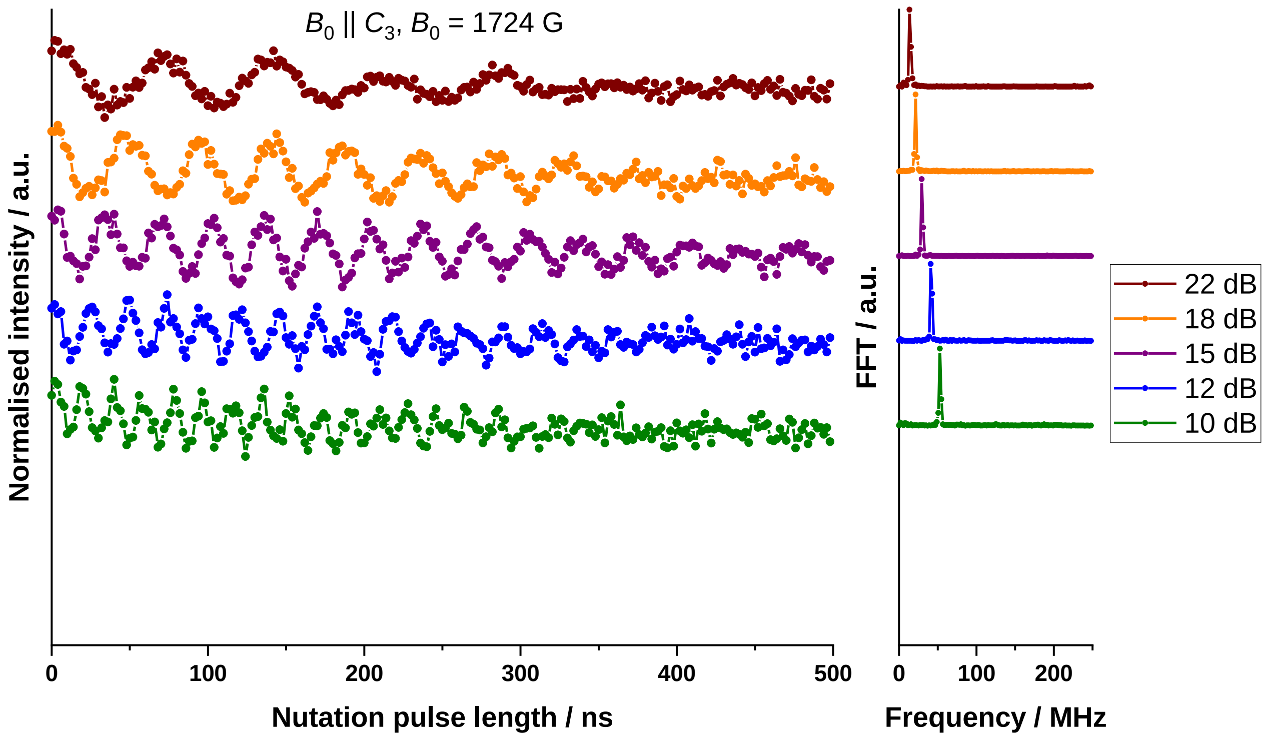

Figure S21 *Left*: Rabi oscillations as a function of microwave power for a crystal of **1** oriented with $B_0 \parallel C_3$ and with B_0 = 1724 G. *Right*: Fourier transform of the Rabi oscillations. The measurements were performed at 8 K.

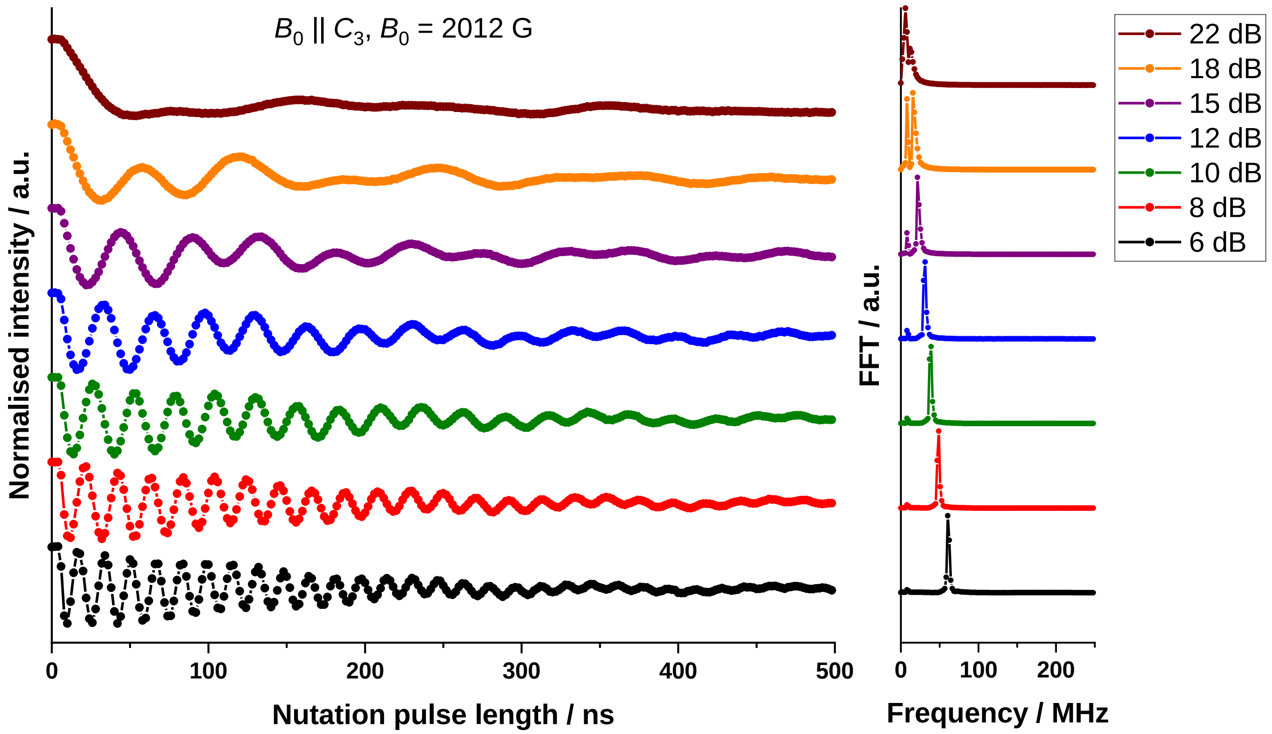

Figure S22 *Left*: Rabi oscillations as a function of microwave power for a crystal of **1** oriented with $B_0 \parallel C_3$ and with B_0 = 2012 G. *Right*: Fourier transform of the Rabi oscillations. The measurements were performed at 8 K.

Table S2. Temperature and magnetic field dependence of T_1 for a single crystal of **1** oriented with $\vec{B} \perp C_3$. The word in the parentheses indicates whether the transition is for a single Yb^{III} site (Single) or for a coupled Yb^{III} site (Coupled).

Temperature	2032 G (Single)	2296 G (Coupled)	2426 G (Coupled)	2590 G (Single)
2 K	31.5 ± 0.7 ms	795 ± 17 μs	1251 ± 15 μs	16.5 ± 0.4 ms
3.5 K	3.30 ± 0.02 ms	549 ± 12 μs	739 ± 13 μs	3.14 ± 0.02 ms
5 K	323 ± 2 μs	113 ± 2 μs	134 ± 2 μs	300 ± 2 μs
6.5 K	91.5 ± 0.8 μs	37.7 ± 0.8 μs	44.1 ± 1 μs	83.4 ± 0.8 μs
8 K	17.5 ± 0.03 μs	11.4 ± 0.06 μs	12.3 ± 0.07 μs	17.7 ± 0.02 μs
11 K	2.57 ± 0.01 μs	2.22 ± 0.01 μs	2.37 ± 0.01 μs	3.60 ± 0.005μs
14 K	1.23 ± 0.001μs	787 ± 5 ns	825 ± 7 ns	1.22 ± 0.001 μs
18 K	384 ± 4 ns	322 ± 18 ns	315 ± 15 ns	370 ± 3 ns

Table S3. Temperature and magnetic field dependence of T_m (in ns) for a single crystal of **1** oriented with $\vec{B} \perp C_3$. The word in the parentheses indicates whether the transition is for a single Yb^{III} site (Single) or for a coupled Yb^{III} site (Coupled).

Temperature	2032 G (Single)	2296 G (Coupled)	2426 G (Coupled)	2590 G (Single)
2 K	200 ± 1	219 ± 5	241 ± 5	224 ± 2
3.5 K	200 ± 1	215 ± 5	228 ± 4	222 ± 1
5 K	196 ± 1	209 ± 3	221 ± 3	220 ± 2
6.5 K	194 ± 1	198 ± 2	216 ± 2	217 ± 1
8 K	181 ± 1	181 ± 3	187 ± 3	197 ± 1
11 K	166 ± 1	156 ± 1	169 ± 1	160 ± 1
14 K	113 ± 1	103 ± 1	109 ± 1	109 ± 0.3
18 K	67 ± 1	78 ± 6	-	71 ± 1

Table S4. Temperature and magnetic field dependence of T_1 for a single crystal of **1** oriented with $\vec{B} \parallel C_3$. The word in the parentheses indicates whether the transition is for a single Yb^{III} site (Single) or for a coupled Yb^{III} site (Coupled).

Temperature	1330 G (Single)	1544 G (Coupled)	1724 G (Coupled)	2012 G (Single)
3.5 K	2.37 ± 0.04 ms	1.52 ± 0.01 ms	1.54 ± 0.04 ms	2.54 ± 0.01 ms
5 K	185 ± 11 μs	134 ± 2 μs	127 ± 4 μs	181 ± 1 μs
6.5 K	52.4 ± 6.1 μs	39.1 ± 1.5 μs	31.8 ± 0.9 μs	47.2 ± 0.6 μs
8 K	12.7 ± 0.2 μs	9.26 ± 0.05 μs	9.02 ± 0.11 μs	12.8 ± 0.06 μs
11 K	2.15 ± 0.1 μs	1.72 ± 0.03 μs	1.86 ± 0.1 μs	2.49 ± 0.05 μs
14 K	844 ± 28 ns	-	-	762 ± 0.01 ns

Table S5. Temperature and magnetic field dependence of T_m (in ns) for a single crystal of **1** oriented with $\vec{B} \parallel C_3$. The word in the parentheses indicates whether the transition is for a single Yb^{III} site (Single) or for a coupled Yb^{III} site (Coupled).

Temperature	1330 G (Single)	1544 G (Coupled)	1724 G (Coupled)	2012 G (Single)
6.5 K	139 ± 2	183 ± 6	190 ± 7	190 ± 2
8 K	108 ± 3	141 ± 8	158 ± 10	144 ± 2
11 K	106 ± 2	92 ± 2	65 ± 1	88 ± 1

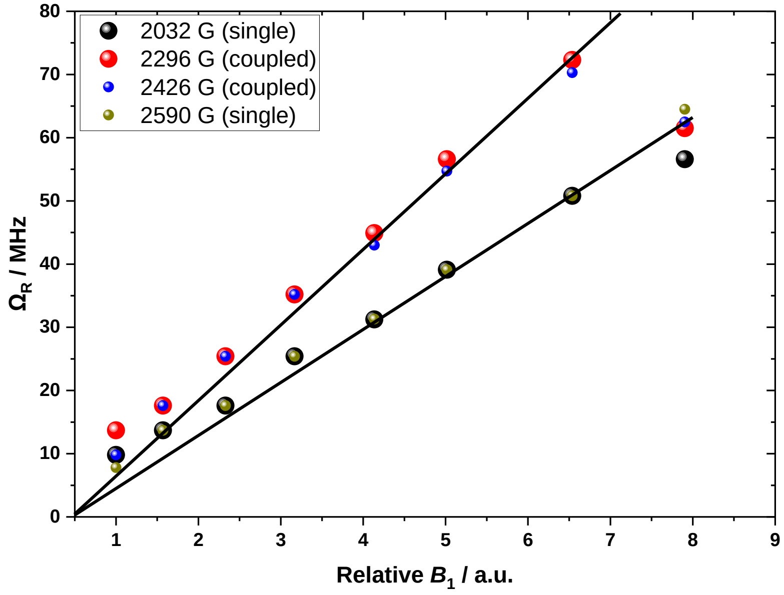

Figure S23. B_1 dependence of the Rabi frequencies obtained at 8 K for selected magnetic field positions on a single crystal of **1** oriented with $\vec{B} \perp C_3$. The word in the parentheses tells whether the transition is for a single Yb^{III} site (Single) or for a coupled Yb^{III} site (Coupled). The black lines are guidelines for the eye illustrating the linear dependence of Ω_R on B_1.

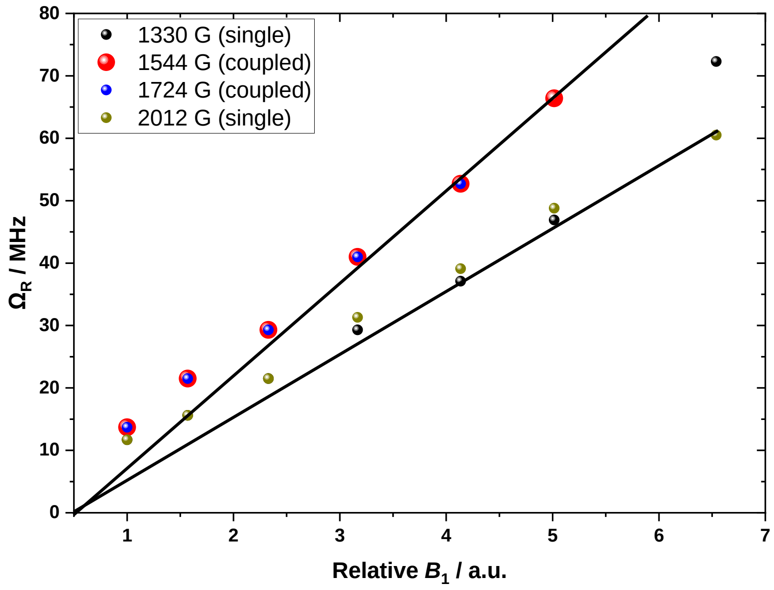

Figure S24. B_1 dependence of the Rabi frequencies obtained at 8 K for selected magnetic field positions on a single crystal of **1** oriented with $\vec{B} \parallel C_3$. The word in the parentheses tells whether the transition is for a single Yb^{III} site (Single) or for a coupled Yb^{III} site (Coupled). The black lines are guidelines for the eye illustrating the linear dependence of Ω_R on B_1.

Table S6. The Rabi frequencies (Ω_R) in MHz at different magnetic field positions and at different microwave attenuations for a single crystal of **1** oriented with $\vec{B} \perp C_3$ measured at 8 K. The word in the parentheses tells whether the transition is for a single Yb^{III} site (Single) or for a coupled Yb^{III} site (Coupled).

Attenuation	Ω_R(2032 G) (Single)	Ω_R(2296 G) (Coupled)	Ratio	Ω_R(2426 G) (Coupled)	Ω_R(2590 G) (Single)	Ratio
4 dB	56.6	61.5	1.09	62.5	64.5	0.97
6 dB	50.8	72.3	1.42	70.3	50.8	1.38
8 dB	39.1	56.6	1.45	54.7	39.1	1.40
10 dB	31.3	44.9	1.44	43.0	31.3	1.37
12 dB	25.4	35.2	1.39	35.2	25.4	1.39
15 dB	17.6	25.4	1.44	25.4	17.6	1.44
18 dB	13.7	17.6	1.28	17.6	13.7	1.28
22 dB	9.8	13.7	1.40	9.8	7.8	1.26

Table S7. The Rabi frequencies (Ω_R) in MHz at different magnetic field positions and at different microwave attenuations for a single crystal of **1** oriented with $\vec{B} \parallel C_3$ measured at 8 K. The word in the parentheses tells whether the transition is for a single Yb^{III} site (Single) or for a coupled Yb^{III} site (Coupled).

Attenuation	Ω_R(1330 G) (Single)	Ω_R(1544 G) (Coupled)	Ratio	Ω_R(1724 G) (Coupled)	Ω_R(2012 G) (Single)	Ratio
6 dB	72.3	-	-	-	60.5	-
8 dB	46.9	66.4	1.42	-	48.8	-
10 dB	37.1	52.7	1.42	52.7	39.1	1.35
12 dB	29.3	41	1.40	41.0	31.3	1.31
15 dB	21.5	29.3	1.36	29.3	21.5	1.36
18 dB	15.6	21.5	1.38	21.5	15.6	1.38
22 dB	11.7	13.7	1.17	13.7	11.7	1.17

Table S8. Eigenvector compositions at B_0 = 0, with $\vec{B} \| C_3$ and the parameters given in the main text.

	$\|\uparrow\uparrow\rangle$	$\|\uparrow\downarrow\rangle$	$\|\downarrow\uparrow\rangle$	$\|\downarrow\downarrow\rangle$
$\langle 1\|$	0.1447844911	0.6921253146	0.6921253146	-0.1447844912
$\langle 2\|$	0.0000000000	-0.7071067812	0.7071067812	0.0000000000
$\langle 3\|$	-0.7071067811	0.0000000000	0.0000000000	-0.7071067813
$\langle 4\|$	-0.6921253147	0.1447844911	0.1447844911	0.6921253145

Table S9. Eigenvector compositions at B_0 = 1 G, with $\vec{B} \| C_3$ and the parameters given in the main text.

	$\|\uparrow\uparrow\rangle$	$\|\uparrow\downarrow\rangle$	$\|\downarrow\uparrow\rangle$	$\|\downarrow\downarrow\rangle$
$\langle 1\|$	0.1385198742	0.6920830659	0.6920830659	-0.1511764012
$\langle 2\|$	0.0000000000	-0.7071067812	0.7071067812	0.0000000000
$\langle 3\|$	-0.6977794747	-0.00839377	-0.00839377	-0.7162142794
$\langle 4\|$	-0.7027916115	0.144743133	0.144743133	0.6813096225

Table S10. Eigenvector compositions at B_0 = 10 G, with $\vec{B} \| C_3$ and the parameters given in the main text.

	$\|\uparrow\uparrow\rangle$	$\|\uparrow\downarrow\rangle$	$\|\downarrow\uparrow\rangle$	$\|\downarrow\downarrow\rangle$
$\langle 1\|$	-0.0857585027	-0.6876032102	-0.6876032102	0.216908114
$\langle 2\|$	0.6082637784	0.0857911455	0.0857911455	0.784407378
$\langle 3\|$	0.0000000000	-0.7071067812	0.7071067812	0.0000000000
$\langle 4\|$	-0.7890884964	0.1408605861	0.1408605861	0.5810816943

Table S11. Eigenvector compositions at B_0 = 100 G, with $\vec{B} \| C_3$ and the parameters given in the main text.

	$\|\uparrow\uparrow\rangle$	$\|\uparrow\downarrow\rangle$	$\|\downarrow\uparrow\rangle$	$\|\downarrow\downarrow\rangle$
$\langle 1\|$	0.1323550629	-0.1295403504	-0.1295403504	0.9741256246
$\langle 2\|$	-0.1156620656	-0.6921956003	-0.6921956003	-0.1683828627
$\langle 3\|$	0.0000000000	-0.7071067812	0.7071067812	0.0000000000
$\langle 4\|$	0.9844310153	-0.0639104729	-0.0639104729	-0.1507530398

Table S12. Eigenvector compositions at B_0 = 1000 G, with $\vec{B}||C_3$ and the parameters in the main text.

	$\|\uparrow\uparrow\rangle$	$\|\uparrow\downarrow\rangle$	$\|\downarrow\uparrow\rangle$	$\|\downarrow\downarrow\rangle$
$\langle 1\|$	-0.0151020391	0.0090584785	0.0090584785	-0.9998038889
$\langle 2\|$	-0.0121513418	-0.7069981924	-0.7069981924	-0.0126276223
$\langle 3\|$	0.0000000000	-0.7071067812	0.7071067812	0.0000000000
$\langle 4\|$	-0.999812119	0.0084557639	0.0084557639	0.0152553861

References

(1) Vesborg, P. C.; Chorkendorff, I.; Brock-Nannestad, T.; Dethlefsen, J. R.; Bendix, J., '*Note: simple means for selective removal of the 365 nm line from the Hg spectrum using Dy*', *Rev Sci Instrum* **2011**, *82*, 096102.

(2) Pedersen, K. S.; Ungur, L.; Sigrist, M.; Sundt, A.; Schau-Magnussen, M.; Vieru, V.; Mutka, H.; Rols, S.; Weihe, H.; Waldmann, O.; Chibotaru, L. F.; Bendix, J.; Dreiser, J., '*Modifying the properties of 4f single-ion magnets by peripheral ligand functionalisation*', *Chem. Sci.* **2014**, *5*, 1650-1660.

(3) Pedersen, K. S.; Dreiser, J.; Weihe, H.; Sibille, R.; Johannesen, H. V.; Sørensen, M. A.; Nielsen, B. E.; Sigrist, M.; Mutka, H.; Rols, S.; Bendix, J.; Piligkos, S., '*Design of Single-Molecule Magnets: Insufficiency of the Anisotropy Barrier as the Sole Criterion*', *Inorganic Chemistry* **2015**, *54*, 7600-7606.
